# RNase H-dependent PCR (rhPCR): improved specificity and single nucleotide polymorphism detection using blocked cleavable primers

**DOI:** 10.1186/1472-6750-11-80

**Published:** 2011-08-10

**Authors:** Joseph R Dobosy, Scott D Rose, Kristin R Beltz, Susan M Rupp, Kristy M Powers, Mark A Behlke, Joseph A Walder

**Affiliations:** 1 Integrated DNA Technologies, Inc., 1710 Commercial Park, Coralville, IA 5224, USA

## Abstract

**Background:**

The polymerase chain reaction (PCR) is commonly used to detect the presence of nucleic acid sequences both in research and diagnostic settings. While high specificity is often achieved, biological requirements sometimes necessitate that primers are placed in suboptimal locations which lead to problems with the formation of primer dimers and/or misamplification of homologous sequences.

**Results:**

*Pyrococcus abyssi *(*P.a*.) RNase H2 was used to enable PCR to be performed using blocked primers containing a single ribonucleotide residue which are activated via cleavage by the enzyme (rhPCR). Cleavage occurs 5'-to the RNA base following primer hybridization to the target DNA. The requirement of the primer to first hybridize with the target sequence to gain activity eliminates the formation of primer-dimers and greatly reduces misamplification of closely related sequences. Mismatches near the scissile linkage decrease the efficiency of cleavage by RNase H2, further increasing the specificity of the assay. When applied to the detection of single nucleotide polymorphisms (SNPs), rhPCR was found to be far more sensitive than standard allele-specific PCR. In general, the best discrimination occurs when the mismatch is placed at the RNA:DNA base pair.

**Conclusion:**

rhPCR eliminates the formation of primer dimers and markedly improves the specificity of PCR with respect to off-target amplification. These advantages of the assay should find utility in challenging qPCR applications such as genotyping, high level multiplex assays and rare allele detection.

## Background

Quantitative PCR (qPCR) is usually performed in real-time mode using fluorescence detection methods. In one commonly used format (the 5'-nuclease assay), qPCR involves three oligonucleotides wherein the forward and reverse primers direct DNA amplification spanning the hybridization site for a third fluorescently labeled oligonucleotide probe. The probe typically contains a fluorescence reporter dye and a quencher. Separation of the reporter and quencher due to cleavage of the probe by the 5'-nuclease activity of the DNA polymerase leads to an increase of fluorescence and a detectable signal [1-3]. Quantitative PCR can also use nucleic acid binding dyes such as SYBR^® ^Green or Eva Green^® ^that increase fluorescence in the presence of double-stranded DNA (dsDNA). Nucleic acid binding dye systems use only two oligonucleotides, the forward and reverse primers, which direct amplification of the target. Once amplification has occurred, the dye binds to the double stranded DNA and generates a fluorescent signal without the need for a third dye-labeled oligonucleotide probe. Dye binding assays are less expensive and are very convenient; however, they are inherently less specific than three-oligonucleotide systems since signal is generated from any amplification event.

Formation of primer-dimers and off-target amplification are common problems in PCR [4-6]. These competing side reactions are a particular problem with low copy number targets due to the high number of cycles required for amplification and in multiplex assays where many different primers must function well together. While "primer-dimers" are often thought to arise from self-amplification of primers due to overlapping 3'-ends, these species can also be generated when there is little apparent complementarity between the primers [7]. More complex oligomeric products of greater length than primer-dimers are also observed in some cases [8]. The formation of primer-dimers can give rise to a false positive signal in dye-binding qPCR assays, and can lead to false negative results by consumption of primers and other reaction components. Several methods can be used to reduce these undesired side reactions, or mitigate their effects. Physical barrier methods can be used to separate reaction components until an elevated temperature is reached [9,10]. Use of a chemically or antibody inactivated "hot-start" polymerase can alleviate mis-priming at low temperature, but at significant additional cost [11-14]. "Nesting" of primers can detect the desired product among the previously amplified PCR products, but this technique is not applicable to qPCR. Melt-curve analysis done as an additional end-point step in dye-binding qPCR assays can help demonstrate assay specificity by revealing the existence of multiple amplicons, but cannot prevent or limit their formation. Often several assays must be designed and empirically tested before one is found that does not result in multiple melt peaks.

A wide variety of approaches have been employed to confer single-base specificity to PCR assays with the goal of detecting single nucleotide polymorphisms (SNPs) [15,16]. Assays have been based on either of two methods: differential amplification of the variant alleles (allele-specific PCR, or ASPCR) or discrimination between the alleles following or concurrent with unbiased amplification of the target sequence. The most common format for detection concurrent with unbiased amplification is the 5'-nuclease assay [1,17]. In that case, a fluorescence-quenched probe, which is degraded by the 5'-nuclease activity of the DNA polymerase, is designed to bind preferentially to the match sequence relative to a mismatch sequence. In order to distinguish effectively between hybridization of an exact match and a single base pair mismatch, relatively short probes, 12-16 bases in length, are needed. To achieve binding of the probe under conditions of the extension reaction with temperatures typically between 55°C and 70°C, modified bases such as locked nucleic acids (LNAs) or pendant groups such as a minor groove binder (MGB) are incorporated into the oligonucleotide to increase the *T_m _*[18-20].

In ASPCR, the SNP is positioned at or near the 3'-end of the primer such that a mismatch with the template inhibits initiation of DNA synthesis. Even with careful optimization of reaction conditions, the success rate is highly variable. Assays can be improved by incorporating modified bases or by introducing a secondary mismatch within the primer [21-24]. The most serious shortcoming of this assay format is that once extension has occurred off of a mismatched target, the primer becomes incorporated in the amplicon. After the newly synthesized strand is copied, the primer forms a perfect match with the template and no further discrimination can be achieved. Even if the efficiency of replication of the template is reduced 100-fold due to the mismatch, there would only be a differential amplification of 6-7 cycles between alleles.

**Figure 1:**
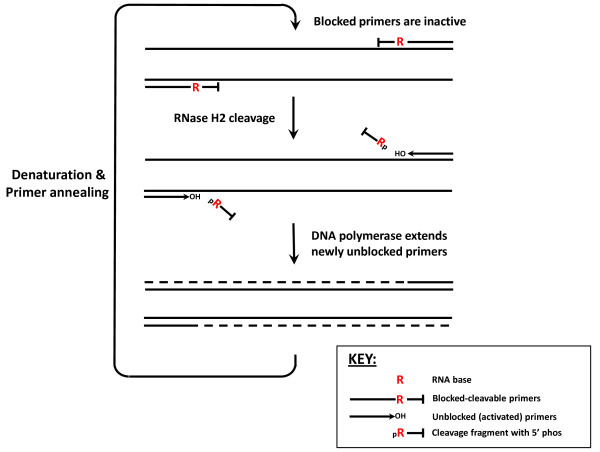
**Coupled reaction scheme for PCR using blocked primers activated by cleavage with RNase H2 (rhPCR)**. PCR primers are designed to be incapable of extension by DNA polymerase and contain a single ribonucleotide residue near the 3'-end. Hybridization of primer to template forms a substrate for RNase H2, which will cleave the primer 5'-to the RNA base leaving a DNA oligonucleotide with a 3'-OH capable of priming DNA synthesis. The assay can be performed using either 2- or 3-step PCR with anneal/extend times as short as 30 seconds.

Here we describe the properties of a thermophilic archaeal RNase H2 enzyme from *Pyrococcus abyssi*, and methods to use this enzyme in a coupled reaction for PCR based assays (RNase H2-dependant PCR or rhPCR) shown schematically in Figure 1. Primers containing a single RNA residue are modified at or near the 3'-end of the oligonucleotide to prevent extension by DNA polymerase. Deblocking and activation of the primers occur upon hybridization to the target DNA sequence and subsequent cleavage by RNase H2. The *Pyrococcus abyssi *(*P.a*.) RNase H2 enzyme has sufficient thermal stability and a high enough turnover rate to perform this function in real time during thermocycling. Cleavage occurs at the 5'-side of the RNA base leaving a DNA oligonucleotide with a 3'-hydroxyl that is competent to function as a primer. *P.a*. RNase H2 has minimal activity at room temperature so that use of this enzyme in rhPCR with blocked primers enables a universal hot start reaction with any thermostable DNA polymerase. Little to no modification in reaction temperatures, cycling times, or analysis procedures is required for inclusion of the RNase H2 enzyme into current end-point PCR and qPCR methods. The requirement for hybridization of the primers to the target sequence for activation prevents template independent reactions such as the formation of primer-dimers. Mismatches at or near the RNA:DNA base pair significantly decrease the efficiency of cleavage by RNase H2, minimizing misamplification of partially homologous sequences. When utilized for the detection of single nucleotide polymorphisms (SNPs), rhPCR was found to be far more sensitive than standard allele-specific PCR. Discrimination between variant alleles is generally greatest when the mismatch is positioned at the RNA:DNA base pair.

## Methods

### Oligonucleotides

Oligonucleotides were synthesized at Integrated DNA Technologies (Coralville, IA) using standard phosphoramidite chemistry. All sequences are listed in Table S1 (Additional file 1).

### Cloning and purification of *P.a*. RNase H2

Cloning, expression, and purification of recombinant *Pyrococcus abyssi *RNase H2 are fully described in the additional methods (Additional file 2). Briefly, the sequence was obtained from public databases [25] and the open reading frame was codon optimized for expression in *E. coli*. A synthetic gene was made and cloned into a shuttle plasmid by the Synthetic Biology group at Integrated DNA Technologies. The gene was transferred to the pET-27b(+) expression vector (Novagen, Madison, WI), which introduced a His-Tag^® ^into the open reading frame. The recombinant protein was expressed in the *E. coli *strain BL21 (DE3). Bacterial lysates were purified using nickel affinity chromatography. Stock solutions of the enzyme were stored in Buffer A (10 mM Tris pH 8.0, 1 mM EDTA, 100 mM NaCl, 0.1% Triton X-100, and 50% glycerol) at -20°C. The enzyme has been stored under these conditions for over 2 years without detectable loss of activity.

### RNase H2 cleavage assays

RNase H2 activity was measured with four duplex substrates, one for each RNA base, having a 14-1-15 design with 14 DNA bases, 1 RNA base, and 15 DNA bases on one strand with complementary DNA bases on the opposite strand. The sequences are shown in Table S1 in the Additional file 1. RNase H2 cleavage reactions were performed with 40 pmoles substrate in Mg Cleavage Buffer (10 mM Tris-HCl pH 8.0, 50 mM NaCl, 4 mM MgCl_2_, 10 μg/mL BSA, 0.01% Triton X-100) for 20 minutes at 70°C. Reactions were stopped with the addition of EDTA to a final concentration of 10 mM. Reaction products were resolved on a 15% polyacrylamide/7 M urea denaturing gel and visualized on a UV transilluminator after staining for 30 min with 1× GelStar™ Nucleic acid stain (Lonza, Basel, Switzerland).

The effect of single base pair mismatches on substrate cleavage by *P.a*. RNase H2 was studied using 34 duplex substrates. The perfect match substrate (S-rC 14-1-15) was a 30 mer with the sequence 5'-CTCGTGAGGTGATGcAGGAGATGGGAGGCG-3', having all complementary DNA bases on the opposite strand. DNA bases are shown in uppercase and the one RNA base is shown in lowercase. The region studied in the mismatch analysis is underlined. The ribo-C containing strand was paired with 33 different mismatch complements where every possible single base mutation was introduced at each of the 11 positions indicated above (positions -5 to +5 relative to the RNA base). Cleavage reactions consisted of 2 pmoles of unlabeled substrate spiked with 40 fmoles of 5'-^32^P-labeled substrate and 0.3 mU of *P.a*. RNase H2. (1 Unit of *P.a*. RNase H2 is the amount of enzyme needed to cleave 1 nmole of S-rC 14-1-15 per minute at 70°C in Mg Cleavage Buffer, and corresponds to approximately 5 fmoles of enzyme.) Reactions were incubated for 20 minutes at 70°C in 20 μL of Mg Cleavage Buffer. Reaction products were separated by PAGE on a 15% polyacrylamide/7 M urea denaturing gel and visualized using a Packard Cyclone™ Storage Phosphor scanner (Packard Biosciences, Meriden, CT). The percentage of cleaved vs. uncleaved substrate was determined using the OptiQuant™ ver. 4.00 software (Packard Biosciences, Meriden, Connecticut).

### *Pyrococcus abyssi *RNase H2 heat stability assays

RNase H2 temperature stability was assessed in triplicate by pre-incubating aliquots of *P.a*. RNase H2 at 95°C for increasing periods of time, ranging from 0 to 90 minutes. Thermal inactivation of the RNase H2 enzymatic activity was detected by reduced substrate cleavage efficiency. Cleavage reactions consisted of 2 pmoles of unlabeled S-rC 14-1-15 spiked with 40 fmoles of 5'-^32^P-labeled substrate and 0.1 mU of the pre-incubated *P.a*. RNase H2 enzyme. Detection and quantification of reaction products were performed as outlined above.

### *Pyrococcus abyssi *RNase H2 temperature dependence studies

The enzymatic activity of *P.a*. RNase H2 was assessed at different reaction temperatures ranging from 30°C to 70°C. Cleavage reactions consisted of 2 pmoles of unlabeled S-rC 14-1-15 spiked with 40 fmoles of 5'-^32^P-labeled substrate and 0.25 mU of *P.a*. RNase H2 in 20 μL of Mg Cleavage Buffer. Reactions were allowed to proceed for 20 minutes and products were separated by PAGE on a 15% polyacrylamide/7 M urea denaturing gel and quantified as described above.

### Optimization of rhPCR primer design and amplification protocols using a synthetic amplicon

Optimization of blocked-cleavable primer design, enzyme concentration, buffer composition, and cycling parameters was done using a synthetic template. Reaction products were examined by separation on a 15% polyacrylamide/7 M urea denaturing gel, stained for 30 min with 1× GelStar™ Nucleic acid stain (Lonza, Basel, Switzerland), and visualized under UV excitation. In addition, the template was amplified in qPCR assays using either SYBR^® ^Green or a dual-labeled fluorescence-quenched hydrolysis probe for detection. The target oligonucleotide that defines this amplicon is a 103mer synthetic sequence that is not homologous to any known gene (see Table S1). Reactions were run on a Roche Lightcycler^® ^480 in 384 well plates using 10 μL reaction volumes. Cycling conditions included an initial 5 minute soak at 95°C, followed by 45 cycles of 10 seconds at 95°C and 30 seconds at 60°C. For some experiments, the dwell time at 60°C was varied between 30, 60, and 120 seconds. Reactions were minimally performed in triplicate.

SYBR^® ^Green reactions (10 μL) consisted of 5 μL of 2× BIO-RAD iQ™ SYBR^® ^Green Supermix (BIO-RAD, Hercules, CA), 200 nM each of the forward and reverse primers, 20 to 2 × 10^6 ^copies of the synthetic oligonucleotide template, with varying amounts of *P.a*. RNase H2. The iQ™ SYBR^® ^Green Supermix contains 3 mM MgCl_2_. 5'-nuclease probe hydrolysis reactions consisted of 0.4 U iTaq DNA polymerase (BIO-RAD, Hercules, CA), 1 μL 10X iTaq™ buffer (BIO-RAD, Hercules, CA), 3 mM MgCl_2_, 800 μM dNTPs, and 200 nM each of the forward and reverse primers and the probe, 20 to 2 × 10^6 ^copies of the synthetic oligonucleotide template, with varying amounts of *P.a*. RNase H2. The quantification cycle number (Cq) was determined using the absolute quantification/2^nd ^derivative method [26].

### Hepatitis C Virus (HCV) Primer-Dimer Studies

Primer-dimer studies employed a HCV amplicon that was previously shown to produce significant primer-dimer artifacts [7]. A 242 base pair HCV target was made as a synthetic gene and cloned in a plasmid by the Synthetic Biology group at Integrated DNA Technologies (Coralville, IA). qPCR assays were performed in triplicate on a Roche Lightcycler^® ^480 in 10 μL reaction volumes with 5 μL of 2× DyNAmo™ SYBR^® ^Green qPCR kit (NEB, Ipswich, MA), 200 nM of each primer, 2 × 10^4 ^copies of the synthetic HCV template plasmid with or without 2 ng rat spinal cord cDNA, and 0 or 2.6 mU of *P.a*. RNase H2. The DyNAmo™ SYBR^® ^Green qPCR reaction mix contains 2.5 mM MgCl_2 _final concentration. Cycling conditions consisted of an initial 5 minute soak at 95°C, followed by 50 cycles of 95°C for 30 seconds and 60°C for 30 seconds. Primer sequences and the sequence of the HCV amplicon are shown in Table S1. Products were separated by PAGE on a 15% non-denaturing polyacrylamide gel, stained for 30 min with 1× GelStar™ Nucleic acid stain (Lonza, Basel, Switzerland), and visualized under UV excitation.

### Studies of the specificity of rhPCR using mammalian cDNA

The performance and specificity of rhPCR with complex nucleic acid samples was first examined using a qPCR assay designed against a human gene target (*HRAS*). Amplification reactions were compared using human cDNA (prepared from HeLa cell total RNA) and rat cDNA (prepared from rat spinal cord total RNA). Blocked-cleavable and control primers that produce a 340 bp amplicon from the human *HRAS *gene (NM_005343) were synthesized (see Table S1). HRAS qPCR assays were performed on a Roche Lightcycler^® ^480 in 10 μL reaction volumes, containing 1 μL *P.a*. RNase H2 (1.3 mU), 5 μL 2× BIO-RAD iQ™ SYBR^® ^Green Supermix (BIO-RAD, Hercules, CA), 2 ng of rat spinal cord cDNA or 2 ng HeLa cDNA, and 200 nM of each primer. Cycle conditions included an initial 5 minute soak at 95°C, followed by 60 or 90 cycles of 10 seconds at 95°C and 90 seconds at 60°C. All reactions were run in triplicate.

### Analysis of the SMAD7 rs4939827 SNP with rhPCR

Reactions were performed on a Roche Lightcycler^® ^480 in 10 μL final volume with 5 μL 2× BIO-RAD iQ™ SYBR^® ^Green Supermix, 200 nM forward and reverse primers and 2 or 20 ng of genomic DNA (GM18562 or GM18537, obtained from the Coriell Institute for Medical Research Cell Repository (http://ccr.coriell.org/)). Reactions were performed in triplicate. One μL (2.6 mU) of *P.a*. RNase H2 in Buffer A or Buffer A without RNase H2 was added to each reaction. Thermal cycling was performed using an initial 5 minute soak at 95°C followed by 45 cycles of 10 seconds at 95°C and 30 seconds at 60°C. Additional experiments were performed varying the cycling parameters and concentration of *P.a*. RNase H2 as outlined in the Results section. Certain studies were performed using up to 100 cycles. All reactions were performed using the same unblocked reverse primer. Forward primers included unmodified allele-specific primers as well as blocked-cleavable primers of different designs. A non-discriminatory unmodified primer served as a control. Cq and ΔCq values were computed as described above.

A subset of studies was performed using DNA from 31 individuals obtained from the Coriell Institute for Medical Research Cell Repository with known genotypes at the SMAD7 rs4939827 locus (Table 1).

**Table 1:** 

C/C homozygotes	C/T heterozygotes	T/T homozygotes
GM18562, GM12874, GM18506, GM18505, GM18503, GM18526, GM18545, GM18558, GM10857, GM12057	GM07029, GM07348, GM10860, GM18563, GM18564, GM18573, GM18576, GM18593, GM18635, GM07055, GM06994	GM18537, GM18561, GM18623, GM18976, GM18992, GM19154, GM18960, GM11881, GM12146, GM12145

## Results

### Recombinant RNase H2 from *Pyrococcus abyssi*

The *rnhb *gene coding for the Type II RNase H from *Pyrococcus abyssi *has been identified previously [27,28]. We produced and purified recombinant *Pyrococcus abyssi *RNase H2 from *E. coli *as outlined in the additional methods (Additional file 2). As expected, the enzyme was found to cleave heteroduplex substrates having a single ribonucleotide comprising any of the four RNA bases. Mass spectrometry analysis confirmed that cleavage occurred on the 5'-side of the RNA residue, yielding one fragment with a free 3'-OH group and a second with a 5'-ribonucleotide phosphate (see Figure S1, Additional file 3). Importantly, single-stranded RNA-containing oligonucleotides were not cleaved or otherwise degraded, demonstrating the absence of any contaminating nuclease activity in the enzyme preparation.

Magnesium requirements were optimized for *P.a*. RNase H2 by examining the dependence of the cleavage rates on Mg^2+ ^concentration for the single rC containing 30mer heteroduplex substrate S-rC 14-1-15 at 70°C. Maximum activity was achieved around 4 mM Mg^2+ ^and high levels of activity were seen in the range of 1 to 10 mM Mg^2+^, similar to other RNase H2 enzymes characterized [28-30]. The enzyme maintained over 90% activity at 2 mM Mg^2+^, and only dropped to 61% of optimal activity in 1 mM Mg^2+ ^(see Figure S2, Additional file 4). No cleavage was observed in the absence of divalent cations. As with other Type II RNase H enzymes [29,30], *P.a*. RNase H2 also has the ability to utilize Mn^2+ ^and Co^2+ ^in place of Mg^2+ ^(data not shown).

The 5'-reaction product formed upon cleavage with RNase H2 has the structure of a normal primer used to initiate PCR reactions: the sequence is entirely DNA and ends in a 3'-hydroxyl group. Reaction conditions for *P.a*. RNase H2 are compatible with the buffers commonly employed in PCR (Mg^2+ ^concentrations, pH, etc.). If the enzyme has sufficient thermal stability and a high enough turnover rate, then it should be possible to perform primer cleavage/activation in real time during PCR.

### Thermal stability and temperature dependence of *Pyrococcus abyssi *RNase H2

**Figure 2:**
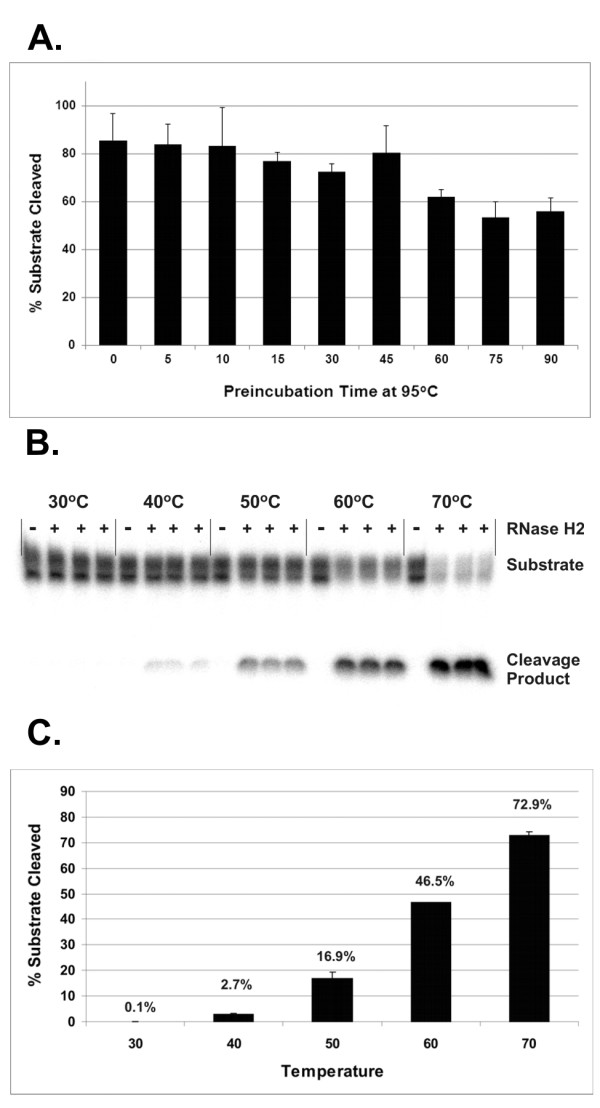
**Thermal stability and temperature dependence of *P.a*. RNase H2 activity**. A. Aliquots of *P.a*. RNase H2 were pre-incubated at 95°C for the indicated times and then tested for activity with the heteroduplex substrate S-rC 14-1-15 (labeled with ^32^P). Cleavage reactions were allowed to proceed for 20 minutes at 70°C. Reaction products were separated by denaturing PAGE and visualized by phosphorimaging. Percent cleavage of the substrate (Y-axis) is shown plotted against pre-incubation time at 95°C (X-axis). Assays were run in triplicate for each pre-incubation time point. B. ^32^P-labeled S-rC 14-1-15 was incubated in the absence or presence of 0.25 mU of recombinant *P.a*. RNase H2 for 20 minutes at 30°C, 40°C, 50°C, 60°C, or 70°C. Reactions were stopped with the addition of EDTA and cleavage products were separated by denaturing PAGE and visualized by phosphorimaging. A gel image is shown. Cleavage reactions were run in triplicate and no enzyme controls were run once. C. The phosphor gel image from panel B above was quantified and triplicate data points averaged. The percent cleavage of substrate (Y-axis) is shown plotted against reaction temperature (X-axis).

To assess the thermal stability of *P.a*. RNase H2, aliquots of the enzyme were incubated at 95°C in Mg Cleavage Buffer for times ranging from 0 to 90 minutes before performing a cleavage activity assay. The *P.a*. RNase H2 protein retained nearly full activity after heating at 95°C for 45 minutes (Figure 2A). After 90 minutes incubation at 95°C, the enzyme retained 2/3 of its original activity. These results demonstrate that *P.a*. RNase H2 is highly resistant to heat inactivation and will tolerate thermocycling conditions similar to those used in PCR.

The temperature-dependence of the activity of *P.a*. RNase H2 was studied by comparing the ability of the enzyme to cleave the substrate S-rC 14-1-15 at different temperatures between 30°C and 70°C (Figures 2B and 2C). Minimal cleavage (< 1%) of the substrate was observed at 30°C, indicating that *P.a*. RNase H2 is effectively inactive at room temperature. In contrast, the *P.a*. RNase H2 enzyme cleaved over 70% of the total substrate at 70°C. The enzyme retained 64% relative activity at 60°C and 23% relative activity at 50°C. Below 50°C, the enzyme rapidly lost activity. These results demonstrate that *P.a*. RNase H2 is highly active throughout the range of temperatures commonly used in PCR and is only minimally active at ambient temperatures.

### Effect of single base pair mismatches on substrate cleavage by *P.a*. RNase H2

**Figure 3:**
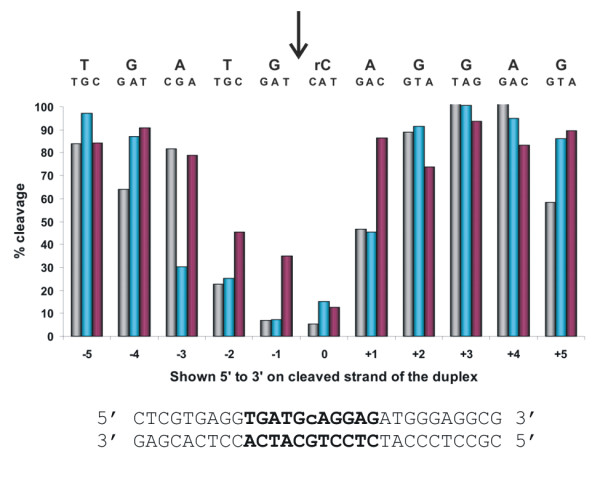
**Differential cleavage of mismatched substrates by *P.a*. RNase H2**. A 30mer oligonucleotide S-rC 14-1-15 having a single RNA base (rC) was paired with its perfect match DNA complement (shown) or oligonucleotides having a single base mismatch at one of the 11 positions identified in bold. Every possible mismatch at these locations was tested. DNA bases are uppercase and RNA bases are lowercase. ^32^P-labeled duplexes were incubated with RNase H2 at 70°C. Reaction products were separated by PAGE, and the extent of cleavage of the substrate was quantified by phosphorimaging. The percent cleavage of each of the mismatched duplexes relative to the perfect match (= 100%) are plotted. The sequence of the invariant RNA-containing top strand is shown above the plot with each mismatch base aligned above its associated data point in the bar graph. The site of cleavage by RNase H2 is indicated by the arrow.

To define the specificity of *P.a*. RNase H2 with respect to single base mismatches, a series of 30mer heteroduplex substrates having a single ribo-C base were synthesized with S-rC 14-1-15 serving as the perfect match control. The ribo-C containing top strand of S-rC 14-1-15 was hybridized to 33 different DNA bottom strands which comprised every possible single base mutation at the RNA base and at flanking positions 1-5 on either side of the RNA residue. Cleavage assays were performed and results are shown in Figure 3. Outside positions "-3 to +1", the presence of a mismatch had little or no effect on cleavage by the enzyme. As has been seen with other RNase H2 enzymes [31,32], the greatest effect was observed for mismatches flanking the cleavage site (positions "-1" and "0"). A mismatch at these locations inhibited cleavage by approximately 10-fold. Within the limits of detection using autoradiography, the only products observed with mismatched substrates resulted from cleavage on the 5'-side of the RNA residue.

### rhPCR assays using *P.a*. RNase H2

A 103 base synthetic oligonucleotide template was used as an artificial amplicon for optimization of primer design for rhPCR. Initial testing of *P.a*. RNase H2 using radiolabeled substrates indicated that 8 to 10 base pairs of duplex DNA 5'-to the RNA residue and 4 to 5 base pairs of DNA duplex 3'-to the RNA base were necessary for optimal cleavage (data not shown). In rhPCR (Figure 1), the 5'-cleavage product must be a functional primer. This ensures that more than 10 bases of DNA are always present on the 5'-side of the RNA residue. The synthetic amplicon system was employed to test the structural requirements on the 3'-side of the RNA residue for efficient cleavage by RNase H2 during thermocycling.

A single unmodified forward (For) PCR primer was paired with a set of blocked-cleavable reverse (Rev) primers having different numbers of DNA residues 3'-to the RNA base. The Rev primers all contained the same 22 base primer domain on the 5'-end followed by a single ribonucleotide (rU) plus 2, 3, 4, 5, or 6 DNA bases complementary to the target and ended in a 3'-terminal dideoxy-cytosine (ddC). The ddC residue blocks primer extension; primer function is activated by RNase H2 cleavage 5'-to the ribonucleotide residue. After 45 cycles (10 seconds at 95°C followed by 30 seconds at 60°C), reaction products were separated by PAGE. No PCR products were produced using blocked primers in the absence of RNase H2, indicating that the Rev primers were inactive and did not spontaneously cleave to give rise to an active species under PCR conditions (see Figure S3, Additional file 5). In the presence of RNase H2, amplification products were not seen for the 2-DNA or 3-DNA primers whereas the correct amplification product was observed using the 4-DNA, 5-DNA, and 6-DNA primers, consistent with earlier studies of substrate cleavage by the enzyme under steady state conditions. Identical results were observed when the 3'-terminus was blocked with a C3 propanediol spacer instead of ddC.

**Figure 4:**
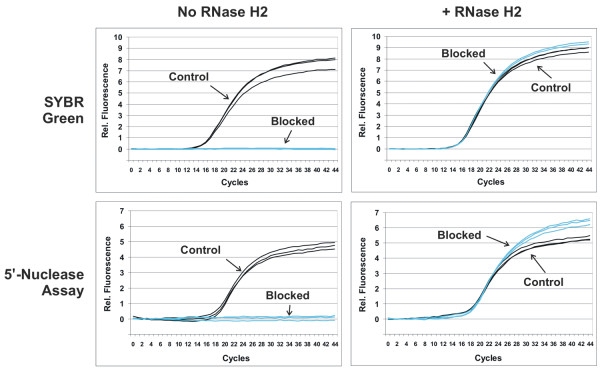
**Real-time amplification plots comparing the performance of blocked-cleavable primers (rhPCR) with unmodified primers**. Unmodified control primers (black) and "rDDDDx" blocked-cleavable primers (blue) were used to detect a synthetic oligonucleotide amplicon in real-time qPCR format. Amplification reactions were performed in the absence of RNase H2 (left panels) or in the presence of 2.6 mU of RNase H2 (right panels). Detection was done using either SYBR^® ^Green (top panels) or a 5'-nuclease assay with a dual-labeled hydrolysis probe (bottom panels).

To examine the relative efficiency of rhPCR, real-time qPCR assays were performed with the blocked-cleavable primers using either SYBR^® ^Green or the 5'-nuclease assay for detection. Figure 4 compares results for amplification reactions using blocked cleavable "rDDDDx" primers (RNA+4-DNA residues followed by a C3 spacer "x") versus control unmodified primers. As before, reactions done without RNase H2 (Figure 4, left panels) showed no detectable amplification with the blocked primers. In the presence of RNase H2 (Figure 4, right panels), blocked and control unmodified primers showed identical performance. The overall shape of the amplification plots, absolute increases in relative fluorescence, and Cq values (detection cycle point for quantification) were indistinguishable. Reactions were identical whether SYBR^® ^Green or the 5' nuclease assays were employed for detection. In these studies, the target was present in high amounts (2 × 10^6 ^copies). The blocked and unmodified primers showed identical amplification efficiency throughout a standard curve down to 20 copies of target (data not shown). These results demonstrate that RNase H2 cleavage of blocked primers can proceed with sufficient speed to achieve efficient amplification and that rhPCR is compatible with real-time detection using either dye binding or probe hydrolysis assays. With an anneal/extension time of 30 seconds, 2.6 mU of *P.a*. RNase H2 per 10 μL is required to achieve identical amplification efficiency between the blocked-cleavable primers and the control primers. A reciprocal relationship exists between the duration of the anneal/extension step and the concentration of RNase H2 required (data not shown). For example, with an anneal/extension time of 60 seconds, 1.3 mU of *P.a*. RNase H2 per 10 μL is needed to achieve parity between the blocked-cleavable and unmodified primers.

### Elimination of primer-dimer formation using rhPCR

Primer-dimer and other misamplification events are a common problem in PCR and are a particular issue in SYBR^® ^Green qPCR assays where false-positive signal detected from misamplified products can occur. Sequence analysis, careful design of PCR primers, and use of hot start methods can reduce the frequency of primer-dimer and misamplification events, but by necessity some assays must be designed in the contexts of sequences that are not ideal. This problem is further magnified in the setting of multiplex PCR where several different primer pairs must function well together. Due to the low level of activity of *P.a*. RNase H2 at room temperature (Figures 2B and 2C), use of this enzyme in rhPCR should result in a primer-based hot-start reaction. Moreover, amplification requires that the primers anneal and form a suitable heteroduplex substrate for RNase H2 cleavage, which should impart greater specificity to the start of the reaction than is provided by hybridization of the primer to the target alone. Taken together, these two factors should mitigate the formation of primer-dimers.

**Figure 5:**
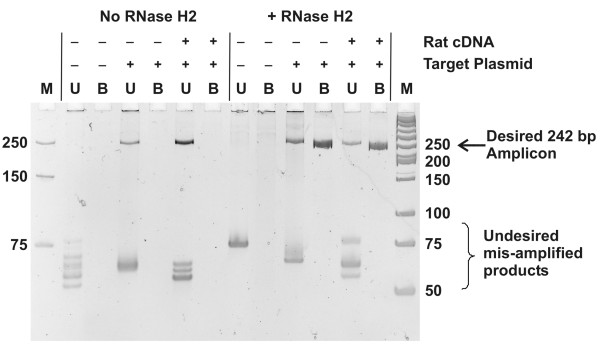
**Elimination of primer-dimer artifacts with rhPCR**. A 242 bp HCV amplicon known to produce primer-dimer artifacts was used to compare the specificity of unmodified control (U) and "rDDDDx" blocked-cleavable (B) primers. M = marker lane (bp, double-stranded). PCR assays were run without (left) or with (right) RNase H2 and included reactions with primers alone, primers plus the synthetic HCV target, or primers and the synthetic HCV target with high complexity rat cDNA. Reaction products were separated by non-denaturing PAGE, fluorescently stained, and visualized by UV excitation.

To test this possibility, we studied the use of rhPCR to prevent primer-dimer formation in a Hepatitis C Virus (HCV) 1b V154 system (Accession #EU660388) where the need to position primers in specific areas of the viral sequence that are highly conserved between strains resulted in an assay extremely prone to the formation of primer-dimers [7]. A synthetic 242 bp HCV amplicon was cloned into a plasmid and an unmodified DNA polymerase (DyNAmo™) was used for PCR. Control DNA primers were those previously described [7]. The corresponding blocked-cleavable rhPCR primers were synthesized by adding to the 3'-end of the sequence a single ribonucleotide, 4 DNA bases complementary to the target, and a 3'-C3 spacer ("rDDDDx"). Sequences of the amplicon and the primers are shown in Table S1 Additional file 1. PCR was performed with zero or 2 × 10^4 ^copies of the HCV target plasmid, with or without RNase H2. In some reactions, 2 ng of rat spinal cord cDNA was added to provide a complex nucleic acid environment. Products were examined using PAGE and results are shown in Figure 5. As expected, use of the unmodified primers resulted in multiple amplified species much smaller than the expected 242 bp amplicon which were independent of input target. The "primer-dimer" products dominated the reaction and variable levels of the desired HCV amplicon were produced. Consistent with primer-dimer formation being a stochastic event, the precise pattern varied with each reaction (and between replicates, data not shown). Amplification using the blocked-cleavable primers was dependent on the presence of RNase H2. In contrast to standard PCR, rhPCR using blocked-cleavable primers produced the correct 242 bp amplicon when the HCV target was present and no amplified products were seen when the target was absent. Use of blocked-cleavable primers in the rhPCR format totally eliminated the formation of primer-dimer artifacts in this system.

### Improved specificity of rhPCR with complex nucleic acid samples

**Figure 6:**
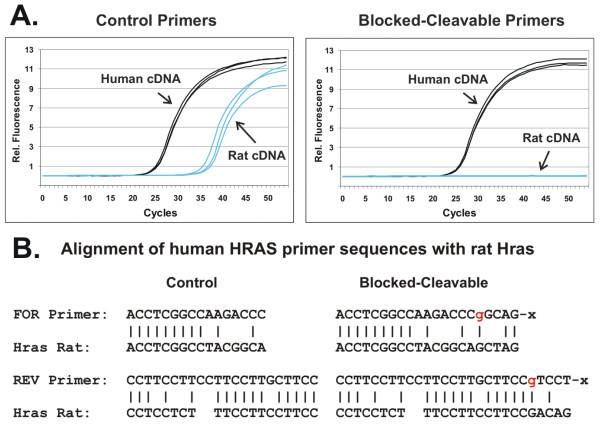
**Increased specificity of rhPCR with complex DNA samples**. A PCR assay specific for the human *HRAS *gene was used to compare the specificity of "rDDDDx" blocked-cleavable primers (rhPCR) with unmodified control primers to amplify the desired sequences in human cDNA versus mismatched sequences in rat cDNA. A. Amplification plots are shown for SYBR^® ^Green qPCR assays run with unmodified control primers (left panel) or blocked-cleavable primers (right panel). Reactions with human cDNA (HeLa) are shown in black and reactions with rat cDNA (spinal cord) are shown in blue. The concentration of RNase H2 was 1.3 mU per 10 μL and the anneal/extension time was 90 seconds. B. The human *HRAS*-specific amplification primers are shown aligned with the homologous sequence in the rat *Hras *gene. Unmodified control primers are shown on the left and blocked-cleavable primers are shown on the right. DNA bases are black uppercase and RNA bases are red lowercase. "x" is a propanediol C3 spacer.

The HCV example demonstrated that rhPCR improves reaction specificity by preventing primer-dimer formation (a template independent event), even in a system highly prone to this problem. A qPCR assay specific for the human v-Ha-ras Harvey rat sarcoma viral oncogene homolog (*HRAS*) was used to test whether rhPCR could improve reaction specificity and reduce misamplification of sequences closely related to the target. *HRAS *amplification reactions were done using cDNA prepared from human HeLa cells or from rat spinal cord. Using the control primers (Figure 6A, left panel), signal was observed with human cDNA, the true target for which the primers were designed, at a Cq of 25.9 cycles and for rat cDNA at a Cq of 35-38 cycles. In contrast, with rhPCR using "rDDDDx" blocked-cleavable primers (Figure 6A, right panel), signal was observed in human cDNA at a Cq of 26.2 cycles and no signal was detected out to 55 cycles using rat cDNA. Thus the control primers showed a false positive signal with a ΔCq of ~11 cycles between human cDNA (true positive) and rat cDNA (false positive) while the blocked-cleavable primers gave signal only with the true target. Reactions were performed again using 90 cycles to maximize the chance that mispriming events with the blocked primers could be detected. A false positive signal for rat cDNA was eventually observed at 79 cycles (a ΔCq of 53, 42 cycles later than the control primers).

The human *HRAS *control and blocked-cleavable primers are aligned against the corresponding rat *Hras *sequences in Figure 6B. In spite of multiple mismatches present in both the forward and reverse control primers, amplification from rat cDNA was observed with only an 11 cycle delay compared to the perfect match. As a result of the presence of mismatches around the site of cleavage (the ribonucleotide base), both primers form poor substrates for RNase H2, conferring an additional level of specificity to rhPCR and giving greatly improved performance compared to the control primers.

### Detection of single base pair mismatches with rhPCR

**Figure 7:**
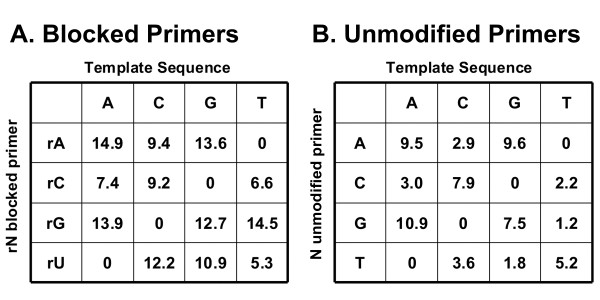
**Mismatch discrimination using blocked-cleavable primers with an RNA:DNA base-pair mismatch versus standard unmodified allele-specific PCR primers**. Four synthetic 103 base oligonucleotide targets were employed where a single base was varied (A, C, G, or T) within the primer binding site. For each DNA target, a single common For primer was paired with four different "rDDDDx" blocked-cleavable Rev primers, varying the RNA base (A), or with four unmodified allele-specific primers terminating in a different 3'-DNA base (B). Amplification reactions were performed in real-time mode using SYBR^® ^Green detection; all reactions were run in triplicate. ΔCq values are shown in the tables and represent the difference between the Cq value for the allele-specific primer for each template and the Cq value for the perfect match template. The concentration of RNase H2 was 1.3 mU per 10 μL.

The ability of rhPCR to discriminate single base mismatches was first studied using the synthetic 103mer target sequence described above. A single unmodified For primer was paired with a set of 4 "rDDDDx" blocked-cleavable Rev primers, one for each possible RNA base. A set of 4 matching targets were synthesized placing each possible DNA base at the position complementary to the RNA residue. The 16 pairwise combinations of these primers and targets were tested for efficiency in a SYBR^® ^Green qPCR assay wherein 4 combinations were a perfect match and the other 12 combinations represented every possible mismatch pairing. For comparison, a set of 4 traditional allele-specific PCR primers were tested where the 3'-terminal base was at the site of the polymorphism under interrogation. ΔCq values (the difference between the Cq value for the allele-specific primer for each template and the Cq value for the perfect match template) are shown in Figure 7. Standard errors of the ΔCq values were less than or equal to 0.1 cycles in all cases except when the rA primer was paired with the G template, where the standard error was 0.49 cycles. Allele-specific PCR using the standard unmodified primers showed ΔCq values ranging from 1.2 to 10.9 with an average of 5.4. The rhPCR assay showed a much higher level of discrimination with ΔCq values ranging from 5.3 to 14.9 and an average cycle delay of 10.9. All twelve mismatches were easily detected with ΔCq values greater than 5 cycles. Two-tailed T-tests comparing the ΔCq values of the unmodified versus modified primers revealed highly statistically significant P values for all combinations except rU:T vs. T:T, for which the ΔCq values were nearly identical (5.3 and 5.2, respectively). For the remaining 11 mismatch combinations, the P value was less than 0.0005 except for the rA:G vs. A:G pair where the P value was calculated to be 0.012. The T:G and G:T mismatch pairs showed the lowest ΔCq values with the traditional allele-specific PCR primers as expected (both less than 2 cycles), since these mismatches form the most thermodynamically stable pairing [33]. Interestingly, these pairs showed excellent discrimination in the rhPCR assay with ΔCq values of 10.9 (rU:G) and 14.5 (rG:T), indicating that mismatch discrimination with rhPCR is not based upon thermodynamic stability of the mismatch pair but instead relies upon enzymatic interrogation of the structure at the cleavage site. These results demonstrate the significant improvement that rhPCR offers over standard allele-specific qPCR.

The experiments shown in Figure 7 examined the effects of a mismatch at the RNA base (position "0" as defined in Figure 3). The effects of mismatches at the "-1" and "+1" positions on the rhPCR assay were also studied. For these experiments, blocked-cleavable Rev primers were synthesized where the RNA base was held constant and the DNA base at position "-1" or "+1" was varied. Primers were made for all 4 RNA bases with all 4 DNA bases at these two flanking positions. Complementary oligonucleotide targets were synthesized as before. SYBR^® ^Green qPCR assays were performed and ΔCq values measured between the perfect match and every possible single base mismatch. Results for the 64 primer/target pairs with a mismatch at position "-1" are shown in Figure S4 (Additional file 6) and those with a mismatch at position "+1" are shown in Figure S5 (Additional file 7). Standard errors of the ΔCq values were less than or equal to 0.3 cycles in all cases. Mismatch discrimination varied from 0.8 to 16.1 cycles for the "-1" series, with an average ΔCq value of 7.7. For the "+1" series, ΔCq varied from 0.2 to 13.8 cycles, with an average value of 6.6. Although mismatch discrimination varied with sequence context, overall the greatest specificity was achieved by placing the mismatch opposite to the RNA base (position "0").

### Application of rhPCR to detect single nucleotide polymorphisms in human genomic DNA

A polymorphism in the human *SMAD7 *gene (NM_005904) associated with increased risk of developing colorectal carcinoma (rs4939827) was used as a model system to study the performance of rhPCR for SNP discrimination with genomic DNA. Blocked-cleavable For primers ("rDDDDx") were synthesized for both alleles with the RNA base positioned at the SNP site. The rs4939827 SNP is a C/T variation. The "C-allele" primer will form a rC:G pair for the match and rC:A for the mismatch. The "T-allele" primer will form a rU:A pair for the match and rU:G for the mismatch. These mismatch combinations showed mid-range discrimination in the synthetic amplicon system having ΔCq values of 7.4 and 10.9, respectively (Figure 7A). Unmodified allele-specific primers were also synthesized where the SNP site was positioned at the 3'-terminal base or the penultimate residue. A non-discriminatory control primer which ended at the "-1" position immediately 5'-to the SNP was used as a control. This set of For primers was used with a single unblocked Rev primer to perform qPCR using SYBR^® ^Green detection with human genomic DNA as template from known homozygotes for each allele at this locus (C/C or T/T). Results are summarized in Table 2. Standard errors were all less than or equal to 0.6 cycles. The unmodified allele-specific primers showed almost no difference between the matched and mismatched targets whether the SNP site was located at the 3'-terminus or the penultimate residue (ΔCq values ranged from 0.5 to 2.1). In contrast, blocked-cleavable primers with the RNA base positioned opposite the SNP site showed a large delay in amplification between the matched and the mismatched alleles; average ΔCq values between the mismatched and matched targets were 12.1 and 12.6 cycles for the "C-allele" and "T-allele" primers, respectively. A 2-tailed T-test evaluation of these results showed that this was a highly statistically significant improvement compared to the unmodified primers (P < 0.005 for both the C and T alleles).

**Table 2:** Mismatch discrimination for various primer designs at the SMAD7 rs4939827 SNP locus

3'-Primer Sequences	Cq values		
	
	(T/T)	(C/C)	ΔCq
...AA	26.0	26.0	0.0
...AAC	28.0	26.0	2.0
...AAT	25.8	26.7	0.9
...AACA	28.2	26.1	2.1
...AATA	25.6	26.1	0.5
...AAC**a**GGAC-x	27.9	26.7	1.3
...AAT**a**GGAC-x	26.9	26.4	0.5
...AA**c**AGGA-x	38.7	26.6	12.1
...AA**u**AGGA-x	27.9	40.5	12.6
...A**a**CAGG-x	36.4	27.5	8.8
...A**a**TAGG-x	26.2	28.8	2.6

Amplification reactions were performed in real-time mode using SYBR^® ^Green detection and either homozygous C/C or homozygous T/T human genomic DNA. Reactions were run in triplicate; average Cq values are shown. ΔCq represents the difference between the Cq value for the mismatched reaction minus the Cq value for the matched reaction. Sequences at the 3'-end of the primers are shown. DNA bases are uppercase and RNA bases are bold lowercase. The C3 spacer blocking group is indicated by "x". The location of the mismatch in the primer compared to the target nucleic acid is underlined.

Studies using the synthetic amplicon system suggested that placing the SNP site opposite the RNA base (position "0") generally gave the highest mismatch discrimination; however, when the SNP site was placed at positions "-1" or "+1" the ΔCq was also very favorable in some cases. To determine the optimal position of the SNP site within the blocked-cleavable primer in the sequence context of the SMAD7 rs4939827 SNP, primers were synthesized for both the C and T alleles placing the SNP site at the "-1" and "+1" positions relative to the RNA base. As with the synthetic template, placing the SNP at the RNA base (position "0") gave the best results. Blocked-cleavable primers with the SNP at the "-1" position performed very poorly with ΔCq values of less than 2 (Table 2). These results were not statistically different from results obtained with the unmodified primers. Placing the SNP site at the "+1" position gave intermediate results with ΔCq values of 8.8 and 2.6 for the "C-allele" and "T-allele" primers, respectively. These results represented a statistically significant improvement over the unmodified primers but reflected decreased specificity when compared with blocked-cleavable primers having the mismatch placed opposite the RNA residue (P < 0.05 for both alleles).

In all of these experiments, thermocycling was performed using a 60°C anneal/extension temperature. Standard unmodified allele-specific primers may be more dependent upon using an optimized reaction temperature than the rhPCR method, so the experiments were repeated with anneal/extension temperatures ranging from 60°C to 70°C. Results with the unmodified primers were slightly better at elevated anneal temperatures, but in no case did the ΔCq between the matched and mismatched targets exceed 4 cycles (data not shown). Experiments performed using the blocked-cleavable primers also showed little improvement in mismatch discrimination by increasing the reaction temperature above 60°C.

In traditional qPCR and PCR reactions, it is occasionally necessary to employ a primer annealing temperature lower than 60°C due to sequence context constraints imposed by the natural nucleic acid targets. To determine whether the lower enzymatic activity of *P.a*. RNase H2 seen at temperatures below 60°C would affect the efficiency of rhPCR or mismatch discrimination, amplification reactions were repeated with the SMAD7 rs4939827-specific blocked-cleavable primers at annealing temperatures of 55°C or 50°C (see Table S2 in Additional file 8). Only a small decrease in amplification efficiency of rhPCR was observed at 50°C with no significant effect on mismatch discrimination. Thus, in spite of the reduction of activity of the *P.a*. RNase H2 enzyme as the reaction temperature is lowered from 60°C to 50°C (see Figure 2C), the efficiency and specificity of rhPCR remains remarkably stable throughout this temperature range.

### Use of rhPCR in genotyping

**Figure 8:**
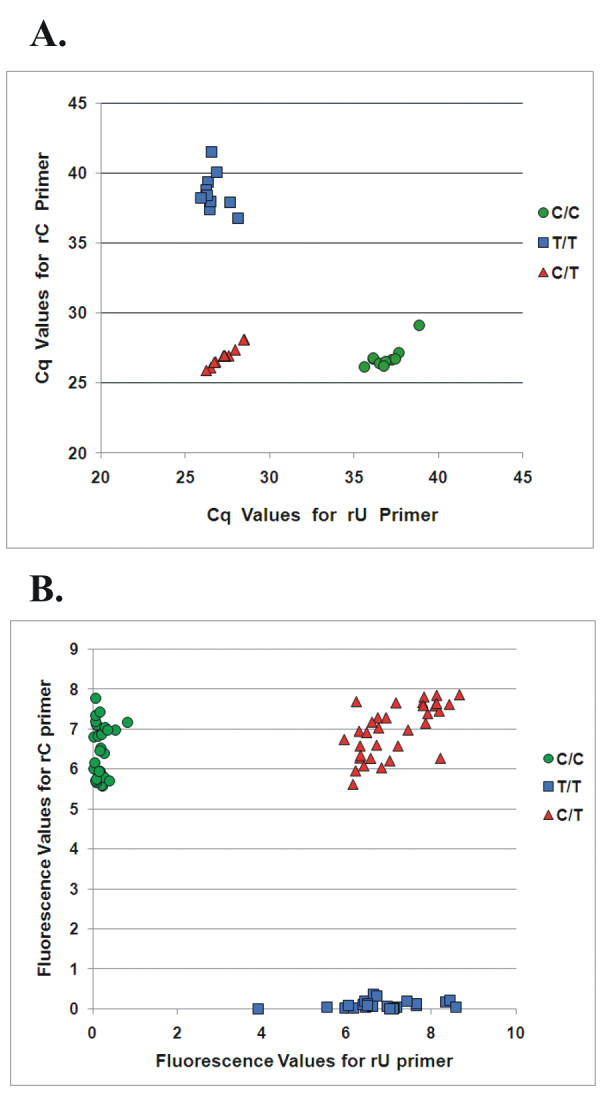
**Application of rhPCR to determine genotype for 31 individuals at the SMAD7 rs4939827 SNP Locus**. Human DNA samples from 31 individuals comprising 10 C/C homozygotes (circles), 10 T/T homozygotes (squares), and 11 C/T heterozygotes (triangles) were obtained from the Coriell Cell Repository and genotypes were determined using rhPCR. Reactions were run in triplicate using 2 ng of genomic DNA with SYBR^® ^Green detection. Separate reactions using unmodified non-discriminatory control primers were performed to ensure that the amount of template was approximately equal in each case. A. Reactions were run in real-time mode for 45 cycles. Cq values for triplicate reactions were averaged and plotted according to the primer employed (rC vs. rU). B. Reactions were run in end-point mode for 35 cycles. The raw fluorescence measurements from each reaction are shown and plotted according to the primer employed (rC vs. rU).

A collection of 31 human DNA samples of known identity at the SMAD7 rs4939827 SNP locus were obtained from the Coriell Repository. This set comprised 10 C/C homozygotes, 10 T/T homozygotes, and 11 C/T heterozygotes. These 31 samples were tested in the rhPCR assay as described above using "rDDDDx" blocked-cleavable primers with the SNP positioned at the RNA base (position "0") in a blinded fashion using 2 ng input genomic DNA and 2.6 mU of the *P.a*. RNase H2 enzyme. The assays were first run in real-time mode using SYBR^® ^Green detection. Results are shown in Figure 8A. All 31 human DNA samples were easily stratified into 3 classes (homozygote C/C, homozygote T/T, and heterozygote C/T) and each genotype was correctly identified.

The rhPCR assay was clearly effective at discriminating these 3 genotypes when run with the benefit of the high precision offered by real-time detection. For high throughput applications, it is more efficient to process plates in batch format and read fluorescence in an end-point mode. The above experiment was therefore repeated running the reaction for 35 cycles and fluorescence was measured at this point; raw fluorescence values were plotted for each sample and are shown in Figure 8B. Each DNA sample was run in triplicate, so the fluorescence measurements include 93 data points. Once again, all 31 human DNA samples (93 PCR reactions) were easily stratified into 3 classes (homozygote C/C, homozygote T/T, and heterozygote C/T) and each genotype was correctly identified. As more amplification cycles are performed, signal from mismatched targets will eventually increase. At 40 cycles the correct genotypes of all samples were still easily identified, but by 45 cycles the boundaries between homozygote and heterozygote samples began to merge (data not shown). By limiting the reaction to 40 cycles or less (with an input of 2 ng of genomic DNA), rhPCR can be used for genotyping applications in an end-point format.

## Discussion

### Primer Cleavage Dependent PCR

Several coupled reaction schemes have been proposed for PCR in which a hybridization dependent primer activation step is linked to primer extension. In the pyrophosphorolysis-activated polymerization (PAP) assay [34,35], a blocked 3'-terminal nucleotide is cleaved by attack of pyrophosphate (reverse of the polymerization reaction). For this to occur efficiently, high concentrations of pyrophosphate are required which may inhibit some polymerases. The range of blocking groups that can be accommodated at the 3'-terminus is very limited. A 3'-terminal dideoxynucleotide has been utilized in most studies [34,35]. Of the four bases, only dideoxy-C can be readily incorporated using standard methods of oligonucleotide synthesis, limiting widespread use of this technique.

A coupled PCR assay has been proposed in which a blocked primer is cleaved after hybridization to the target sequence by a nicking restriction endonuclease [36]. A restriction enzyme that has an asymmetric recognition sequence or that cuts only one strand at a hemimethylated site would be required to avoid cleavage of the template. To our knowledge, this reaction scheme has never been demonstrated experimentally. In any event, the requirement that the restriction enzyme recognition sequence be located near the 3'-end of the primer would severely limit the use of this method.

Use of both RNase H1 and RNase H2 to effect primer cleavage in a coupled PCR assay has been reported previously in the patent literature but minimally characterized [37,38]. Unlike the Type II RNase H enzymes, Type I enzymes will not cleave a substrate having a single RNA residue. At least 3 consecutive RNA residues are required, and 4 for a high level of catalytic activity [39]. Thus, use of a Type I RNase H in rhPCR would require that the primer have at least four consecutive RNA residues. This adds substantially to the cost and complexity of the synthesis of the primer and increases its susceptibility to degradation. The cleaved primer would terminate in two or more RNA residues which can inhibit primer extension and these RNA residues would be incorporated into the amplicon. Sagawa *et al*. [37] suggested that the specificity of Type II RNases H would be similar to that of a restriction enzyme and that cleavage, and hence amplification, would be completely prevented if there was a mismatch at the RNA:DNA base pair within the duplex formed between the primer and the template. Although this is not true, as seen in the present study, coupling RNase H2 cleavage to primer extension can be used to greatly boost the specificity of PCR.

### Use of *P.a*. RNase H2 in rhPCR

The thermostability and temperature dependence of *P.a*. RNase H2 makes it well suited for use in rhPCR. The activity of the enzyme is unaffected by heating at 95°C for 45 minutes (Figure 2A). Critically, this level of resistance to heat inactivation is sufficient for use in PCR, even for reactions requiring an extended number of cycles and a sustained initial incubation at 95°C. To demonstrate this explicitly, *P.a*. RNase H2 was pre-cycled in PCR buffer for 80 cycles (without primers, dNTPs, or template), then all reaction components were added and rhPCR was performed (data not shown). The reaction efficiency was unchanged, indicating that the enzyme's thermal stability is sufficient to remain active throughout the range of use expected for all PCR applications. A further advantage of *P.a*. RNase H2 is that it is essentially inactive at temperatures below 30°C (Figure 2B and 2C). This effectively confers a hot start to reactions done using blocked-cleavable primers and any thermostable DNA polymerase. In addition, *P.a*. RNase H2 has sufficient activity at 50°C to support rhPCR, permitting the reaction to be performed throughout a broad temperature range (Table S2, Additional file 8).

Like other Type II RNase H enzymes, *P.a*. RNase H2 retains a high level of activity at concentrations of Mg^2+ ^as low as 1 mM [28-30], enabling rhPCR to be performed at all magnesium concentrations typically employed in PCR. The enzyme is able to cleave heteroduplex substrates with a single ribonucleotide comprising any of the four bases. Cleavage also occurs at a riboA:deoxyU base pair (not shown), allowing the uracil-N-glycosylase (UNG) sterilization method to be used in rhPCR assays. Although reaction rates are slightly affected by the identity of the RNA base and the flanking DNA sequence, a single concentration of the enzyme and set of reaction conditions generally can be used regardless of the sequence of the target. The efficiency of primer cleavage is principally determined by the structure of the primer 3'-to the RNA base and the degree of complementarity present near the scissile linkage.

It is important to note that non-specific hydrolysis of the RNA linkage cannot lead to primer activation. Water catalyzed hydrolysis and enzymatic cleavage by contaminating single-strand specific ribonucleases both lead to the formation of a cyclic 2'-3'-phosphodiester at the 3'-terminus. This group blocks the 3'-end of the oligonucleotide and prevents primer extension. Spontaneous hydrolysis of the cyclic phosphodiester gives a mixture of 2'- and 3'-phosphate monoesters; enzymatic hydrolysis yields exclusively the 3'-phosphate. In either case, primer extension remains blocked. Background cleavage of the ribonucleotide linkage is problematic only if it occurs to such an extent that the amount of the primer is substantially depleted. Although divalent cations (including Mg^2+^) can facilitate water catalyzed hydrolysis of RNA containing oligonucleotides, especially at elevated temperatures [40], non-enzymatic degradation of primers containing a single RNA residue under thermocycling conditions used in PCR is negligible. If there is significant contamination of a sample with single-stranded ribonucleases, inhibitors such as human placental RNase inhibitor can be included in the reaction mixture as they do not affect the activity of RNase H enzymes. In our experience, this has not been necessary.

### Recognition of Substrates having Base-Pair Mismatches by Type II RNase H Enzymes

RNase H2 plays an important role in the removal of RNA residues misincorporated into DNA due either to incomplete removal of RNA primers used to initiate DNA synthesis or polymerase errors [41-45]. Consistent with its role in DNA repair, Type II RNase H enzymes are also able to cleave substrates where there is an RNA:DNA base pair mismatch, but at a rate reduced compared to the corresponding perfect duplex [31,32,46-48]. For *P.a*. RNase H2, the rate of the reaction is decreased by about 10-fold (Figure 3). A decrease in rate of similar magnitude is seen with a mismatch on the 5'-side of the cleavage site (position "-1"). Mismatches at the "-3", "-2", and "+1" positions gave rise to smaller reductions in the cleavage rate. Outside of this region, effects of a base pair mismatch were negligible. In all cases, the only products observed by mass spectrometry, and by electrophoresis using radiolabeled substrates, reflected cleavage on the 5'-side of the RNA residue. More detailed kinetic studies of the effects of mismatches on cleavage rates are in progress.

### Enhanced specificity of rhPCR

Coupling cleavage by RNase H2 to primer extension in rhPCR leads to greater specificity both with respect to template independent mispriming events (e.g., primer-dimer formation) and unwanted amplification of related sequences. The formation of primer-dimers is prevented even in assays that are very prone to this side reaction (Figure 5). This feature of rhPCR should be particularly beneficial in multiplex assays. The specificity of the assay with respect to misamplification of homologous sequences is far greater than can be achieved by PCR using unmodified primers. When there are mismatches over or neighboring the RNase H2 cleavage sites of both primers, the ΔCq values observed are extremely large. For the *HRAS *gene, the ΔCq between the rat and human sequences was greater than 50 cycles. This high degree of specificity should be very useful for the detection of low levels of heterologous DNA in xenogeneic transplant models (e.g., human tumors grown in a mouse host) and in other instances where there are related targets having closely spaced variations in sequence. In SNP detection, where it is necessary to exploit the effect of a single base pair mismatch on cleavage by RNase H2, the assay also shows far greater discrimination than can be achieved with standard allele-specific PCR.

### Use of rhPCR for Genotyping

Several groups have employed RNase H in SNP discrimination assays using unbiased amplification of the target sequence linked to cleavage of an RNA-containing probe. Harvey, Han, and colleagues described the use of a thermostable RNase H1 enzyme in genotyping assays where cleavage of a fluorescence-quenched probe having four sequential RNA bases was used to discriminate base identity [47]. The assay was coupled to PCR and cleavage of the probe occurred in real time during thermocycling, as in a 5'-nuclease assay. The Type II RNase H enzymes from *Chlamydia pneumonia *(*C.p*.) and from *Thermus thermophilus *(*T.th*.) have been used for SNP detection where a Molecular Beacon having a single RNA residue specific for the mutation site was cleaved following PCR in an end-point assay [32,48,49].

In rhPCR the discrimination between variant alleles relies on differential amplification of the matched and mismatched target sequences. The specificity of the assay is generally greatest when the RNA residue of the primer is placed over the SNP site. In a model system using "rDDDDx" blocked-cleavable primers, all 12 possible base pair mismatches were readily detected (Figure 7A). The average ΔCq was 10.9 using the same set of reaction conditions in each assay. With unmodified allele-specific primers, the average ΔCq was only 5.4.

The application of rhPCR to genotyping of genomic DNA samples was investigated with the SMAD7 rs4939827 (C/T) SNP locus. Individuals homozygous for the T/T allele are at increased risk for the development of colon cancer. With unmodified allele-specific primers, there was almost no discrimination between the two alleles (Table 2), precluding the use of traditional ASPCR for genotyping at this locus. With rhPCR, the two alleles were easily distinguished. Using "rDDDDx" primers with the RNA residue positioned over the SNP site, the ΔCq between matched and mismatched targets was approximately 12 for both the "T-allele" and "C-allele" specific primers. The three genotypes (C/C, C/T, and T/T) could be distinguished unambiguously. In a blinded study of 31 DNA samples representing different individuals, all were correctly identified. The assay was robust with both real-time and end-point modes of detection (Figure 8).

In studies with both a synthetic template (Figures S4 and S5, Additional files 6 and 7) and the SMAD7 SNP locus (Table 2), rhPCR primers with the mutation site located 5'-to the RNA base (position "-1") were less discriminatory than primers where the RNA base was placed over the SNP site. In the case of the SMAD7 locus, rhPCR primers with the SNP site at the "-1" position provided almost no differentiation between the two alleles, similar to unmodified allele-specific primers. At first, it might seem that placing the mutation site on the 5'-side of the RNA base would be optimal, providing discrimination both at the RNase H2 cleavage step and at the initiation of DNA synthesis. In contrast, with the RNA base opposite the mutation site (position "0"), the primer forms a perfect match to the template after RNase H2 cleavage, offering no opportunity for further distinction between the two alleles. However, in the former case, if primer cleavage and extension do occur on a mismatched template, the alternate allele is incorporated into the extension product and a new amplicon is created which is now a perfect match to the primer. As a result, a 10-fold decrease in the cleavage rate by RNase H2 can contribute at most a 3-4 cycle increase in the Cq value. On the other hand, if the RNA base is positioned opposite the mutation site, the mismatched template should be replicated faithfully. When this occurs, the effect of the mismatch becomes amplified with each cycle and produces a much greater increase in the value of ΔCq. For convenience, Additional file 9 contains a merged set of all of the additional files.

### Other applications for thermostable RNase H2 in molecular biology

RNase H2 can also be used to increase the specificity of DNA ligation assays (data not shown). Oligonucleotide ligation assays (OLAs) employ two oligonucleotides (an acceptor oligonucleotide with a reactive 3'-hydroxyl group and a donor oligonucleotide with a 5'-phosphate) which are designed to hybridize adjacent to each other on a complementary target nucleic acid so that DNA ligase can join the two fragments [50-54]. The formation of this new longer species is detectable by a variety of means including PCR and fluorescent bead capture. An added degree of specificity for SNP detection relies upon the ability of DNA ligase to distinguish between a perfect match and mismatched base pair at or near the site of ligation. Donor and/or acceptor oligonucleotides can be designed that are modified to prevent ligation and contain an internal RNA residue near the ligation site. Blocking groups on the acceptor oligonucleotide useful to inhibit ligation are the same as those used to prevent primer extension. As with the blocked-cleavable primers used in rhPCR, cleavage of the blocked-cleavable OLA oligonucleotide will result in a free 3'-hydroxyl which can function as an acceptor for a 5'-phosphate during ligation. Cleavage of the donor oligonucleotide by RNase H2 will result in a 5'-ribophosphate, which will also function efficiently in ligation [55]. This coupled reaction scheme could be employed to improve the specificity of OLAs for nucleic acid detection or genotyping.

## Conclusions

In summary, we describe here a PCR method (rhPCR) that couples activation of blocked-cleavable primers by *P.a*. RNase H2 with amplification. The new rhPCR method simplifies primer design by eliminating the formation of primer-dimers and markedly enhances the specificity of PCR with respect to off-target amplification of closely related sequences. There is no change in workflow and use of a less expensive non-hot start DNA polymerase may actually result in a reduction in assay costs. In addition to its use in genotyping, the assay should find utility in improving the function of highly multiplexed assays where many different primers must work well together without unwanted interactions (primer-dimers, etc.), in rare allele detection where the added specificity of the blocked-cleavable primers enables detection of a desired mutant allele in the face of increasingly large amounts of wild type DNA (manuscript in preparation), and in library construction for Next Generation DNA Sequencing methods to reduce contamination of libraries with primer dimer "blank reads".

## Competing interests

Patent applications have been filed relating to the technologies described herein which are assigned to Integrated DNA Technologies, Inc., (IDT). JAW is both a shareholder and employee of IDT, which offers oligonucleotides and reagents for sale similar to some of the compounds described in this manuscript. All other authors are employed by IDT but do not personally own any shares or equity in IDT. IDT is not a publicly traded company.

## Authors' contributions

JRD, SDR, MAB, and JAW conceived of the study, participated in its design, coordination, and data analysis, and drafted the manuscript. JRD carried out the molecular biology studies and enzyme characterization. KRL and KMP participated in the molecular biology and enzyme characterization studies. SMR produced the recombinant RNase H2 enzyme preparations. All authors read and approved the final manuscript.

## Supplementary Material

Additional File 1 **Table S1. Synthetic oligonucleotide sequences employed in this study**. Click here for file

Additional File 2 **Supplemental Methods: Cloning and characterization of *Pyrococcus abyssi *RNase H2**. Click here for file

Additional File 3 **Figure S1. Identification of RNase H2 cleavage products by mass spectrometry**. The synthetic oligonucleotide substrates shown were examined before and after cleavage by recombinant *Pyrococcus abyssi *RNase H2 using electrospray ionization mass spectrometry (ESI-MS). Mass spectra and measured masses are shown to the left. Substrates and reaction products with calculated molecular weights are shown to the right. DNA bases are indicated in black upper case and RNA bases are indicated in red lower case letters. Click here for file

Additional File 4 **Figure S2. Mg^2+ ^dependence of *P.a*. RNase H2 activity**. ^32^P-labeled substrate S-rC 14-1-15 was incubated in the absence or presence of 0.25 mU of recombinant *P.a*. RNase H2 for 20 minutes at 70°C in Mg Cleavage Buffer (10 mM Tris-HCl pH 8.0, 50 mM NaCl, 10 μg/mL BSA, 0.01% Triton X-100) with varying concentrations of MgCl_2 _as indicated. Reactions were stopped with the addition of EDTA and cleavage products were separated by denaturing PAGE and visualized by phosphorimaging. The phosphor gel image was quantified and the percent cleavage of substrate (Y-axis) is shown plotted against Mg^2+ ^concentration (X-axis). Click here for file

Additional File 5 **Figure S3. Optimization of primer design for rhPCR**. Design of blocked-cleavable primers for use in rhPCR was optimized using a 103 base synthetic oligonucleotide target. A single unmodified Forward (For) primer was used with different blocked-cleavable Reverse (Rev) primers and compared for their relative ability to prime a PCR assay. Blocked-cleavable Rev primers used the same sequence as the unmodified control Rev primer, with the addition of a rU base, and were serially extended by adding 2, 3, 4, 5, or 6 DNA bases 3'-to the ribonucleotide. All blocked primers ended in a ddC residue. Following 45 cycles of PCR, products were separated by denaturing PAGE, fluorescently stained and visualized by UV excitation. M = oligo size markers (bases). Click here for file

Additional File 6 **Figure S4. Mismatch discrimination using rhPCR with the mismatch positioned at the "-1" position relative to the RNA base**. Sixteen synthetic oligonucleotide targets were employed where the base complementary to the single RNA residue in the blocked-cleavable primers was fixed (A, C, G, or T) and the base paired opposite position "-1" immediately 5'-to the RNA base in the primer was varied (A, C, G, or T). Likewise a set of 16 "rDDDDx" blocked-cleavable primers was employed where the RNA base was fixed (rA, rC, rG, or rU) and the base at the "-1" position was varied (A, C, G, or T). The target sequence and primers were otherwise the same as in Figure S3, except that the control non-discriminatory primer was one base shorter on the 3'-end. Assay conditions and calculations of ΔCq values were the same as in Figure 7 in the manuscript. All reactions were run in triplicate. Click here for file

Additional file 7 **Figure S5. Mismatch discrimination using rhPCR with the mismatch positioned at the "+1" position relative to the RNA base**. Sixteen synthetic oligonucleotide targets were employed where the base complementary to the single RNA residue in the blocked-cleavable primers was fixed (A, C, G, or T) and the base paired opposite position "+1" immediately 5'-to the RNA base in the primer was varied (A, C, G, or T). Likewise a set of 16 "rDDDDx" blocked-cleavable primers was employed where the RNA base was fixed (rA, rC, rG, or rU) and the base at the "+1" position was varied (A, C, G, or T). The target sequence and primers were otherwise the same as in Figure S3. Assay conditions and calculations of ΔCq values were the same as in Figure 7 in the manuscript. All reactions were run in triplicate. Click here for file

Additional File 8 **Table S2. Efficiency of rhPCR at different anneal/extend temperatures. **Amplification reactions were run in standard format (10 L reactions with 2.6 mU P.a. RNase H2) using 2-step PCR with anneal/extend temperatures of 50oC, 55oC, and 60oC.  The SMAD7 SNP assay and “rDDDDx” blocked-cleavable primers were employed, as in Table 2. Click here for file

Additional File 9 **Unified additional materials**. All additional files are merged to improve convenience when saving or printing these data. Click here for file
