# Supplementary material for: RNase H-dependent PCR (rhPCR): improved specificity and single nucleotide polymorphism detection using blocked cleavable primers
Source: BMC Biotechnol. 2011 Aug 10;11:80. doi: 10.1186/1472-6750-11-80 (PMC3224242; doi:10.1186/1472-6750-11-80)
Supplement: Additional File 1 — Table S1. Synthetic oligonucleotide sequences employed in this study. [file 1472-6750-11-80-S1.PDF]

Additional File 1.

Table S1. Synthetic oligonucleotide sequences

Figure #	Name	Sequence
**Figure S1**	S-rC 14-1-15	CTCGTGAGGTGATGcAGGAGATGGGAGGCG
	AS-dC	CGCCTCCCATCTCCTGCATCACCTCACGAG
**Figure 2**	S-rC 14-1-15	CTCGTGAGGTGATGcAGGAGATGGGAGGCG
	AS-dC	CGCCTCCCATCTCCTGCATCACCTCACGAG
**Figure S2**	S-rC 14-1-15	CTCGTGAGGTGATGcAGGAGATGGGAGGCG
	AS-dC	CGCCTCCCATCTCCTGCATCACCTCACGAG
**Figure 3**	S1RC Sense	CTCGTGAGGTGATGcAGGAGATGGGAGGCG
	S-rC 14-1-15 antisense	CGCCTCCCATCTCCTGCATCACCTCACGAG
	S-rG 14-1-15 antisense	CGCCTCCCATCTCCTCCATCACCTCACGAG
	S-rA 14-1-15 antisense	CGCCTCCCATCTCCTTCATCACCTCACGAG
	S-rU 14-1-15 antisense	CGCCTCCCATCTCCTACATCACCTCACGAG
	rC AS mismatch -1 T	CGCCTCCCATCTCCTGTATCACCTCACGAG
	rC AS mismatch -1 A	CGCCTCCCATCTCCTGAATCACCTCACGAG
	rC AS mismatch -1 G	CGCCTCCCATCTCCTGGATCACCTCACGAG
	rC AS mismatch -2 G	CGCCTCCCATCTCCTGCGTCACCTCACGAG
	rC AS mismatch -2 C	CGCCTCCCATCTCCTGCCTCACCTCACGAG
	rC AS mismatch -2 T	CGCCTCCCATCTCCTGCTTCACCTCACGAG
	rC AS mismatch -3 G	CGCCTCCCATCTCCTGCAGCACCTCACGAG
	rC AS mismatch -3 A	CGCCTCCCATCTCCTGCAACACCTCACGAG
	rC AS mismatch -3 C	CGCCTCCCATCTCCTGCACCACCTCACGAG
	rC AS mismatch -4 T	CGCCTCCCATCTCCTGCATTACCTCACGAG
	rC AS mismatch -4 A	CGCCTCCCATCTCCTGCATAACCTCACGAG
	rC AS mismatch -4 G	CGCCTCCCATCTCCTGCATGACCTCACGAG
	rC AS mismatch -5 T	CGCCTCCCATCTCCTGCATCTCCTCACGAG
	rC AS mismatch -5 G	CGCCTCCCATCTCCTGCATCGCCTCACGAG
	rC AS mismatch -5 C	CGCCTCCCATCTCCTGCATCCCCTCACGAG
	rC AS mismatch +1 C	CGCCTCCCATCTCCCGCATCACCTCACGAG
	rC AS mismatch +1 A	CGCCTCCCATCTCCAGCATCACCTCACGAG
	rC AS mismatch +1 G	CGCCTCCCATCTCCGGCATCACCTCACGAG
	rC AS mismatch +2 A	CGCCTCCCATCTCATGCATCACCTCACGAG
	rC AS mismatch +2 T	CGCCTCCCATCTCTTGCATCACCTCACGAG

	rC AS mismatch +2 G	**CGCCTCCCATCTCGTGCATCACCTCACGAG**
	rC AS mismatch +3 G	**CGCCTCCCATCTGCTGCATCACCTCACGAG**
	rC AS mismatch +3 T	**CGCCTCCCATCTTCTGCATCACCTCACGAG**
	rC AS mismatch +3 A	**CGCCTCCCATCTACTGCATCACCTCACGAG**
	rC AS mismatch +4 C	**CGCCTCCCATCCCCTGCATCACCTCACGAG**
	rC AS mismatch +4 A	**CGCCTCCCATCACCTGCATCACCTCACGAG**
	rC AS mismatch +4 G	**CGCCTCCCATCGCCTGCATCACCTCACGAG**
	rC AS mismatch +5 A	**CGCCTCCCATATCCTGCATCACCTCACGAG**
	rC AS mismatch +5 T	**CGCCTCCCATTTCCTGCATCACCTCACGAG**
	rC AS mismatch +5 G	**CGCCTCCCATGTCCTGCATCACCTCACGAG**
**Figure S3**	SynRev 6DrU ddC	**CTGAGCTTCATGCCTTTACTGTuCCCCGA/ddC/**
	SynRev 5DrU ddC	**CTGAGCTTCATGCCTTTACTGTuCCCCG/ddC/**
	SynRev 4DrU ddC	**CTGAGCTTCATGCCTTTACTGTuCCCC/ddC/**
	SynRev 3DrU ddC	**CTGAGCTTCATGCCTTTACTGTuCCC/ddC/**
	SynRev 3DrU ddC	**CTGAGCTTCATGCCTTTACTGTuCC/ddC/**
	SynTemp AAG	**AGCTCTGCCCAAAGATTACCCTGACAGCTAAGTGGCAGTGGAAGTTGGCCTC AGAAGTAGTGGCCAGCTGTGTGTCGGGGAACAGTAAAGGCATGAAGCTCAG**
	SynFor unblocked	**AGCTCTGCCCAAAGATTACCCTG**
	SynRev non-discrimin	**CTGAGCTTCATGCCTTTACTGT**
	SynAmp Probe	**FAM-TTCTGAGGCCAACTTCCACTGCCACTTA-IBFQ**
**Figure 4**	SynFor rA C3 blocked	**AGCTCTGCCCAAAGATTACCCTGaCAGC-x**
	SynRev 4DrU C3	**CTGAGCTTCATGCCTTTACTGTuCCCC-x**
	SynTemp AAG	**AGCTCTGCCCAAAGATTACCCTGACAGCTAAGTGGCAGTGGAAGTTGGCCTC AGAAGTAGTGGCCAGCTGTGTGTCGGGGAACAGTAAAGGCATGAAGCTCAG**
	SynFor unblocked	**AGCTCTGCCCAAAGATTACCCTG**
	SynRev non-discrimin	**CTGAGCTTCATGCCTTTACTGT**
	SynAmp Probe	**FAM-TTCTGAGGCCAACTTCCACTGCCACTTA-IBFQ**
**Figure 5**	Control HCV For	**GCAGAAAGCGTCTAGCCATGGCGTTA**
	Control HCV Rev	**GCAAGCACCCTATCAGGCAGTACCACAA**
	HCV 4DrG For	**GCAGAAAGCGTCTAGCCATGGCGTTAgTATG-x**
	HCV 4DrG Rev	**GCAAGCACCCTATCAGGCAGTACCACAAgGCCT-x**
	HCV Amplicon	**GCAGAAAGCGTCTAGCCATGGCGTTAGTATGAGTGTCGTGCAGCCTCCAGGA CCCCCCCTCCCGGGAGAGCCATAGTGGTCTGCGGAACCGGTGAGTACACCGG AATTGCCAGGACGACCGGGTCCTTTCTTGGACTAAACCCGCTCAATGCCTGG AGATTTGGGCGTGCCCCCGCGAGACTGCTAGCCGAGTAGTGTTGGGTCGCGA AAGGCCTTGTGGTACTGCCTGATAGGGTGCTTGC**
**Figure 6**	Control HRAS For	**ACCTCGGCCAAGACCC**

	Control HRAS Rev	CCTTCCTTCCTTCCTTGCTTCC
	HRAS 4D rG For	ACCTCGGCCAAGACCCgGCAG-x
	HRAS 4D rG Rev	CCTTCCTTCCTTCCTTGCTTCCgTCCT-x
**Figure 7**	SynRev 4DrU C3	CTGAGCTTCATGCCTTTACTGTuCCCC/3SpC3/
	SynRev 4DrA C3	CTGAGCTTCATGCCTTTACTGTaCCCC/3SpC3/
	SynRev 4DrC C3	CTGAGCTTCATGCCTTTACTGTcCCCC/3SpC3/
	SynRev 4DrG C3	CTGAGCTTCATGCCTTTACTGTgCCCC/3SpC3/
	SynRev -T unblocked	CTGAGCTTCATGCCTTTACTGTT
	SynRev -A unblocked	CTGAGCTTCATGCCTTTACTGTA
	SynRev -C unblocked	CTGAGCTTCATGCCTTTACTGTC
	SynRev -G unblocked	CTGAGCTTCATGCCTTTACTGTG
	SynFor unblocked	AGCTCTGCCCAAAGATTACCCTG
	SynRev non-discrimin	CTGAGCTTCATGCCTTTACTGT
	SynTemp AAG	AGCTCTGCCCAAAGATTACCCTGACAGCTAAGTGGCAGTGGAAGTTGGCCTC AGAAGTAGTGGCCAGCTGTGTGTCGGGGAACAGTAAAGGCATGAAGCTCAG
	SynTemp ATG	AGCTCTGCCCAAAGATTACCCTGACAGCTAAGTGGCAGTGGAAGTTGGCCTC AGAAGTAGTGGCCAGCTGTGTGTCGGGGTACAGTAAAGGCATGAAGCTCAG
	SynTemp AGG	AGCTCTGCCCAAAGATTACCCTGACAGCTAAGTGGCAGTGGAAGTTGGCCTC AGAAGTAGTGGCCAGCTGTGTGTCGGGGGACAGTAAAGGCATGAAGCTCAG
	SynTemp ACG	AGCTCTGCCCAAAGATTACCCTGACAGCTAAGTGGCAGTGGAAGTTGGCCTC AGAAGTAGTGGCCAGCTGTGTGTCGGGGCACAGTAAAGGCATGAAGCTCAG
**Figure S4**	SynRev 4DrU C3	CTGAGCTTCATGCCTTTACTGTuCCCC/3SpC3/
	SynRev 4D CrU C3	CTGAGCTTCATGCCTTTACTGCuCCCC/3SpC3/
	SynRev 4D GrU C3	CTGAGCTTCATGCCTTTACTGGuCCCC/3SpC3/
	SynRev 4D ArU C3	CTGAGCTTCATGCCTTTACTGAuCCCC/3SpC3/
	SynRev 4DrA C3	CTGAGCTTCATGCCTTTACTGTaCCCC/3SpC3/
	SynRev 4D CrA C3	CTGAGCTTCATGCCTTTACTGCaCCCC/3SpC3/
	SynRev 4D GrA C3	CTGAGCTTCATGCCTTTACTGGaCCCC/3SpC3/
	SynRev 4D ArA C3	CTGAGCTTCATGCCTTTACTGAaCCCC/3SpC3/
	SynRev 4DrC C3	CTGAGCTTCATGCCTTTACTGTcCCCC/3SpC3/
	SynRev 4D CrC C3	CTGAGCTTCATGCCTTTACTGCcCCCC/3SpC3/
	SynRev 4D GrC C3	CTGAGCTTCATGCCTTTACTGGcCCCC/3SpC3/
	SynRev 4D ArC C3	CTGAGCTTCATGCCTTTACTGAcCCCC/3SpC3/
	SynRev 4DrG C3	CTGAGCTTCATGCCTTTACTGTgCCCC/3SpC3/
	SynRev 4D CrG C3	CTGAGCTTCATGCCTTTACTGCgCCCC/3SpC3/
	SynRev 4D GrG C3	CTGAGCTTCATGCCTTTACTGGgCCCC/3SpC3/

	SynRev 4D ArG C3	CTGAGCTTCATGCCTTTACTGAgCCCC/3SpC3/
	SynRev Short	CTGAGCTTCATGCCTTTACTG
	SynFor unblocked	AGCTCTGCCCAAAGATTACCCTG
	SynTemp AAG	AGCTCTGCCCAAAGATTACCCTGACAGCTAAGTGGCAGTGGAAGTTGGCCTC<br>AGAAGTAGTGGCCAGCTGTGTGTCGGGGAACAGTAAAGGCATGAAGCTCAG
	SynTemp TAG	AGCTCTGCCCAAAGATTACCCTGACAGCTAAGTGGCAGTGGAAGTTGGCCTC<br>AGAAGTAGTGGCCAGCTGTGTGTCGGGGATCAGTAAAGGCATGAAGCTCAG
	SynTemp CAG	AGCTCTGCCCAAAGATTACCCTGACAGCTAAGTGGCAGTGGAAGTTGGCCTC<br>AGAAGTAGTGGCCAGCTGTGTGTCGGGGACCAGTAAAGGCATGAAGCTCAG
	SynTemp GAG	AGCTCTGCCCAAAGATTACCCTGACAGCTAAGTGGCAGTGGAAGTTGGCCTC<br>AGAAGTAGTGGCCAGCTGTGTGTCGGGGAGCAGTAAAGGCATGAAGCTCAG
	SynTemp ATG	AGCTCTGCCCAAAGATTACCCTGACAGCTAAGTGGCAGTGGAAGTTGGCCTC<br>AGAAGTAGTGGCCAGCTGTGTGTCGGGGTACAGTAAAGGCATGAAGCTCAG
	SynTemp TTG	AGCTCTGCCCAAAGATTACCCTGACAGCTAAGTGGCAGTGGAAGTTGGCCTC<br>AGAAGTAGTGGCCAGCTGTGTGTCGGGGTTCAGTAAAGGCATGAAGCTCAG
	SynTemp CTG	AGCTCTGCCCAAAGATTACCCTGACAGCTAAGTGGCAGTGGAAGTTGGCCTC<br>AGAAGTAGTGGCCAGCTGTGTGTCGGGGTCCAGTAAAGGCATGAAGCTCAG
	SynTemp GTG	AGCTCTGCCCAAAGATTACCCTGACAGCTAAGTGGCAGTGGAAGTTGGCCTC<br>AGAAGTAGTGGCCAGCTGTGTGTCGGGGTGCAGTAAAGGCATGAAGCTCAG
	SynTemp AGG	AGCTCTGCCCAAAGATTACCCTGACAGCTAAGTGGCAGTGGAAGTTGGCCTC<br>AGAAGTAGTGGCCAGCTGTGTGTCGGGGGACAGTAAAGGCATGAAGCTCAG
	SynTemp TGG	AGCTCTGCCCAAAGATTACCCTGACAGCTAAGTGGCAGTGGAAGTTGGCCTC<br>AGAAGTAGTGGCCAGCTGTGTGTCGGGGGTCAGTAAAGGCATGAAGCTCAG
	SynTemp CGG	AGCTCTGCCCAAAGATTACCCTGACAGCTAAGTGGCAGTGGAAGTTGGCCTC<br>AGAAGTAGTGGCCAGCTGTGTGTCGGGGGCCAGTAAAGGCATGAAGCTCAG
	SynTemp GGG	AGCTCTGCCCAAAGATTACCCTGACAGCTAAGTGGCAGTGGAAGTTGGCCTC<br>AGAAGTAGTGGCCAGCTGTGTGTCGGGGGGCAGTAAAGGCATGAAGCTCAG
	SynTemp ACG	AGCTCTGCCCAAAGATTACCCTGACAGCTAAGTGGCAGTGGAAGTTGGCCTC<br>AGAAGTAGTGGCCAGCTGTGTGTCGGGGCACAGTAAAGGCATGAAGCTCAG
	SynTemp TCG	AGCTCTGCCCAAAGATTACCCTGACAGCTAAGTGGCAGTGGAAGTTGGCCTC<br>AGAAGTAGTGGCCAGCTGTGTGTCGGGGCTCAGTAAAGGCATGAAGCTCAG
	SynTemp CCG	AGCTCTGCCCAAAGATTACCCTGACAGCTAAGTGGCAGTGGAAGTTGGCCTC<br>AGAAGTAGTGGCCAGCTGTGTGTCGGGGCCCAGTAAAGGCATGAAGCTCAG
	SynTemp GCG	AGCTCTGCCCAAAGATTACCCTGACAGCTAAGTGGCAGTGGAAGTTGGCCTC<br>AGAAGTAGTGGCCAGCTGTGTGTCGGGGCGCAGTAAAGGCATGAAGCTCAG
**Figure S5**	SynRev 4DrU C3	CTGAGCTTCATGCCTTTACTGTuCCCC/3SpC3/
	SynRev 4D rUA C3	CTGAGCTTCATGCCTTTACTGTuACCC/3SpC3/
	SynRev 4D rUT C3	CTGAGCTTCATGCCTTTACTGTuTCCC/3SpC3/
	SynRev 4D rUG C3	CTGAGCTTCATGCCTTTACTGTuGCCC/3SpC3/
	SynRev 4DrA C3	CTGAGCTTCATGCCTTTACTGTaCCCC/3SpC3/
	SynRev 4D rAA C3	CTGAGCTTCATGCCTTTACTGTaACCC/3SpC3/

	SynRev 4D rAT C3	**CTGAGCTTCATGCCTTTACTGTaTCCC/3SpC3/**
	SynRev 4D rAG C3	**CTGAGCTTCATGCCTTTACTGTaGCCC/3SpC3/**
	SynRev 4DrC C3	**CTGAGCTTCATGCCTTTACTGTcCCCC/3SpC3/**
	SynRev 4D rCA C3	**CTGAGCTTCATGCCTTTACTGTcACCC/3SpC3/**
	SynRev 4D rCT C3	**CTGAGCTTCATGCCTTTACTGTcTCCC/3SpC3/**
	SynRev 4D rCG C3	**CTGAGCTTCATGCCTTTACTGTcGCCC/3SpC3/**
	SynRev 4DrG C3	**CTGAGCTTCATGCCTTTACTGTgCCCC/3SpC3/**
	SynRev 4D rGA C3	**CTGAGCTTCATGCCTTTACTGTgACCC/3SpC3/**
	SynRev 4D rGT C3	**CTGAGCTTCATGCCTTTACTGTgTCCC/3SpC3/**
	SynRev 4D rGG C3	**CTGAGCTTCATGCCTTTACTGTgGCCC/3SpC3/**
	SynFor unblocked	**AGCTCTGCCCAAAGATTACCCTG**
	SynRev non-discrimin	**CTGAGCTTCATGCCTTTACTGT**
	SynTemp AAG	**AGCTCTGCCCAAAGATTACCCTGACAGCTAAGTGGCAGTGGAAGTTGGCCTC AGAAGTAGTGGCCAGCTGTGTGTCGGGGAACAGTAAAGGCATGAAGCTCAG**
	SynTemp AAT	**AGCTCTGCCCAAAGATTACCCTGACAGCTAAGTGGCAGTGGAAGTTGGCCTC AGAAGTAGTGGCCAGCTGTGTGTCGGGTAACAGTAAAGGCATGAAGCTCAG**
	SynTemp AAA	**AGCTCTGCCCAAAGATTACCCTGACAGCTAAGTGGCAGTGGAAGTTGGCCTC AGAAGTAGTGGCCAGCTGTGTGTCGGGAAACAGTAAAGGCATGAAGCTCAG**
	SynTemp AAC	**AGCTCTGCCCAAAGATTACCCTGACAGCTAAGTGGCAGTGGAAGTTGGCCTC AGAAGTAGTGGCCAGCTGTGTGTCGGGCAACAGTAAAGGCATGAAGCTCAG**
	SynTemp ATG	**AGCTCTGCCCAAAGATTACCCTGACAGCTAAGTGGCAGTGGAAGTTGGCCTC AGAAGTAGTGGCCAGCTGTGTGTCGGGGTACAGTAAAGGCATGAAGCTCAG**
	SynTemp ATT	**AGCTCTGCCCAAAGATTACCCTGACAGCTAAGTGGCAGTGGAAGTTGGCCTC AGAAGTAGTGGCCAGCTGTGTGTCGGGTTACAGTAAAGGCATGAAGCTCAG**
	SynTemp ATA	**AGCTCTGCCCAAAGATTACCCTGACAGCTAAGTGGCAGTGGAAGTTGGCCTC AGAAGTAGTGGCCAGCTGTGTGTCGGGATACAGTAAAGGCATGAAGCTCAG**
	SynTemp ATC	**AGCTCTGCCCAAAGATTACCCTGACAGCTAAGTGGCAGTGGAAGTTGGCCTC AGAAGTAGTGGCCAGCTGTGTGTCGGGCTACAGTAAAGGCATGAAGCTCAG**
	SynTemp AGG	**AGCTCTGCCCAAAGATTACCCTGACAGCTAAGTGGCAGTGGAAGTTGGCCTC AGAAGTAGTGGCCAGCTGTGTGTCGGGGGACAGTAAAGGCATGAAGCTCAG**
	SynTemp AGT	**AGCTCTGCCCAAAGATTACCCTGACAGCTAAGTGGCAGTGGAAGTTGGCCTC AGAAGTAGTGGCCAGCTGTGTGTCGGGTGACAGTAAAGGCATGAAGCTCAG**
	SynTemp AGA	**AGCTCTGCCCAAAGATTACCCTGACAGCTAAGTGGCAGTGGAAGTTGGCCTC AGAAGTAGTGGCCAGCTGTGTGTCGGGAGACAGTAAAGGCATGAAGCTCAG**
	SynTemp AGC	**AGCTCTGCCCAAAGATTACCCTGACAGCTAAGTGGCAGTGGAAGTTGGCCTC AGAAGTAGTGGCCAGCTGTGTGTCGGGCGACAGTAAAGGCATGAAGCTCAG**
	SynTemp ACG	**AGCTCTGCCCAAAGATTACCCTGACAGCTAAGTGGCAGTGGAAGTTGGCCTC AGAAGTAGTGGCCAGCTGTGTGTCGGGGCACAGTAAAGGCATGAAGCTCAG**
	SynTemp ACT	**AGCTCTGCCCAAAGATTACCCTGACAGCTAAGTGGCAGTGGAAGTTGGCCTC AGAAGTAGTGGCCAGCTGTGTGTCGGGTCACAGTAAAGGCATGAAGCTCAG**

	SynTemp ACA	**AGCTCTGCCCAAAGATTACCCTGACAGCTAAGTGGCAGTGGAAGTTGGCCTC AGAAGTAGTGGCCAGCTGTGTGTCGGGACACAGTAAAGGCATGAAGCTCAG**
	SynTemp ACC	**AGCTCTGCCCAAAGATTACCCTGACAGCTAAGTGGCAGTGGAAGTTGGCCTC AGAAGTAGTGGCCAGCTGTGTGTCGGGCCACAGTAAAGGCATGAAGCTCAG**
**Table 2**	rs4939827 Rev unblocked	**CTCACTCTAAACCCCAGCATT**
	rs4939827 For non-discr	**CAGCCTCATCCAAAAGAGGAAA**
	rs4939827 For T	**CAGCCTCATCCAAAAGAGGAAAT**
	rs4939827 For C	**CAGCCTCATCCAAAAGAGGAAAC**
	rs4939827 4DrC C3	**CAGCCTCATCCAAAAGAGGAAAcAGGA/3SpC3/**
	rs4939827 4DrU C3	**CAGCCTCATCCAAAAGAGGAAAuAGGA/3SpC3/**
	rs4939827 For TA	**CAGCCTCATCCAAAAGAGGAAATA**
	rs4939827 For CA	**CAGCCTCATCCAAAAGAGGAAACA**
	rs4939827 4DrAC C3	**CAGCCTCATCCAAAAGAGGAAaCAGG/3SpC3/**
	rs4939827 4DrAT C3	**CAGCCTCATCCAAAAGAGGAAaTAGG/3SpC3/**
	rs4939827 4DCrA C3	**CAGCCTCATCCAAAAGAGGAAACaGGAC/3SpC3/**
	rs4939827 4DTrA C3	**CAGCCTCATCCAAAAGAGGAAATaGGAC/3SpC3/**
**Table S2**	rs4939827 Rev unblocked	**CTCACTCTAAACCCCAGCATT**
	rs4939827 For non-discr	**CAGCCTCATCCAAAAGAGGAAA**
	rs4939827 4DrC C3	**CAGCCTCATCCAAAAGAGGAAAcAGGA/3SpC3/**
	rs4939827 4DrU C3	**CAGCCTCATCCAAAAGAGGAAAuAGGA/3SpC3/**
**Figure 8**	rs4939827 Rev unblocked	**CTCACTCTAAACCCCAGCATT**
	rs4939827 For non-discr	**CAGCCTCATCCAAAAGAGGAAA**
	rs4939827 4DrC C3	**CAGCCTCATCCAAAAGAGGAAAcAGGA/3SpC3/**
	rs4939827 4DrU C3	**CAGCCTCATCCAAAAGAGGAAAuAGGA/3SpC3/**

DNA bases are black uppercase. RNA bases are red lowercase. IBFQ is Iowa Black™-FQ dark quencher. FAM is 6-carboxyfluorescein. ddC is dideoxycytosine. SpC3 is a C3 propanediol spacer. Locations of mismatched bases relative to the target nucleic acid are underlined. Sequences are shown 5N to 3N.
