# Supplementary material for: RNase H-dependent PCR (rhPCR): improved specificity and single nucleotide polymorphism detection using blocked cleavable primers
Source: BMC Biotechnol. 2011 Aug 10;11:80. doi: 10.1186/1472-6750-11-80 (PMC3224242; doi:10.1186/1472-6750-11-80)
Supplement: Additional File 2 — Supplemental Methods: Cloning and characterization of Pyrococcus abyssi RNase H2. [file 1472-6750-11-80-S2.PDF]

Additional File 2:

Additional Methods: Cloning and characterization of *Pyrococcus abyssi* RNase H2

Sequences of RNase H2 (*rnhb*) genes

Sequence of the codon optimized synthetic gene employed to express recombinant *Pyrococcus abyssi* RNase H2 protein is provided below. Standard codon usage tables for *E. coli* were used. DNA sequence identity was verified on both strands. Lower case letters represent vector sequences, including a Bam HI site on the 5′-end and a Hind III site on the 3′-end used for cloning the gene into the expression plasmid pET-27b(+) (Novagen, Madison, WI). Upper case letters represent coding sequences of the RNase H2 enzyme. The endogenous ATG start codon of the RNase H2 gene is underlined (translation starts upstream of this site in the vector).

Codon optimized *rnhb* gene from *Pyrococcus abyssi*

```
ggatccgATGAAAGTTGCAGGTGCAGATGAAGCTGGTCGTGGTCCAGTTATTGGTCCGCTGGTT
ATTGTTGCTGCTGTTGTGGAGGAAGACAAAATCCGCTCTCTGACTAAGCTGGGTGTTAAAGACT
CCAAACAGCTGACCCCGGCGCAACGTGAAAAACTGTTCGATGAAATCGTAAAAGTACTGGATGA
TTACTCTGTGGTCATTGTGTCCCCGCAGGACATTGACGGTCGTAAGGGCAGCATGAACGAACTG
GAGGTAGAAAACTTCGTTAAAGCCCTGAATAGCCTGAAAGTTAAGCCGGAAGTTATTTACATTG
ATTCCGCTGATGTTAAAGCTGAACGTTTCGCTGAAAACATTCGCAGCCGTCTGGCGTACGAAGC
GAAAGTTGTAGCCGAACATAAAGCGGATGCGAAGTATGAGATCGTATCCGCAGCCTCTATCCTG
GCAAAAGTTATCCGTGACCGCGAGATCGAAAAGCTGAAAGCCGAATACGGTGATTTTGGTTCCG
GTTACCCGTCTGATCCGCGTACTAAGAAATGGCTGGAAGAATGGTATAGCAAACACGGCAATTT
CCCGCCGATCGTGCGTCGTACTTGGGATACTGCAAAGAAAATCGAAGAAAAATTCAAACGTGCG
CAGCTGACCCTGGACAACTTCCTGAAGCGTTTTCGCAACaagctt
```

Production and purification of *P.a.* RNase H2

Several preparations of the enzyme were made. The following protocol is optimized and represents the large scale prep which provided enzyme for the bulk of the studies performed here. BL21(DE3) bacterial cells were transformed with a pET-27b(+) plasmid containing the *E. coli* codon-optimized *Pyrococcus abyssi* RNase H2 gene cloned into the plasmid at the BamHI/HindIII sites. Two x 1 L bacterial cultures were grown to log phase in selective LB media. RNase H2 protein production was induced with 1 mM IPTG at 37°C for 6 hours. Cells were harvested at 5,000 rpm for 10 minutes in a Beckman JLA 10.500 rotor and frozen overnight at -20°C. Cell paste was thawed and 50 mL of Bugbuster® Protein Extraction Reagent, 50 kU rLysozyme™ (Novagen), and 2500 U RNase-free DNase I (Roche, Mannheim, Germany) were added per liter of original culture. Cell lysate was incubated with rotation at 25°C for 30 minutes. The lysate was centrifuged at 16,000 x g for 30 minutes to pellet insoluble materials, and the soluble supernatant was removed and placed in a fresh tube. DNase I was heat inactivated at 75°C for 15 minutes and insoluble materials were removed by centrifugation at 16,000 x g for 10 minutes. A 4% to 20% SDS-polyacrylamide gel was run with increasing amounts of soluble and insoluble material and Coomassie stained to estimate the quantity of RNase H2 present in both fractions. The heat treatment was found to result in a very effective

first step of purification; this material was further purified by capture using a His•Bind® column. Elution was performed by 2 x 6 volumes of elution buffer containing 200 mM imidazole. A 70% ammonium sulfate precipitation was performed to concentrate the purified protein. SDS-PAGE revealed a single band of the expected molecular weight (27.6 kDa) with little contaminating material. The enzyme was dialyzed into Buffer A (10 mM Tris pH 8.0, 1 mM EDTA, 100 mM NaCl, 0.1 % Triton X-100, and 50% glycerol) and stored at -20°C.
