# Supplementary material for: RNase H-dependent PCR (rhPCR): improved specificity and single nucleotide polymorphism detection using blocked cleavable primers
Source: BMC Biotechnol. 2011 Aug 10;11:80. doi: 10.1186/1472-6750-11-80 (PMC3224242; doi:10.1186/1472-6750-11-80)
Supplement: Additional File 3 — Figure S1. Identification of RNase H2 cleavage products by mass spectrometry. The synthetic oligonucleotide substrates shown were examined before and after cleavage by recombinant Pyrococcus abyssi RNase H2 using electrospray ionization mass spectrometry (ESI-MS). Mass spectra and measured masses are shown to the left. Substrates and reaction products with calculated molecular weights are shown to the right. DNA bases are indicated in black upper case and RNA bases are indicated in red lower case letters. [file 1472-6750-11-80-S3.PDF]

**Additional File 3:**

**Duplex S1-rC No RNase H2**

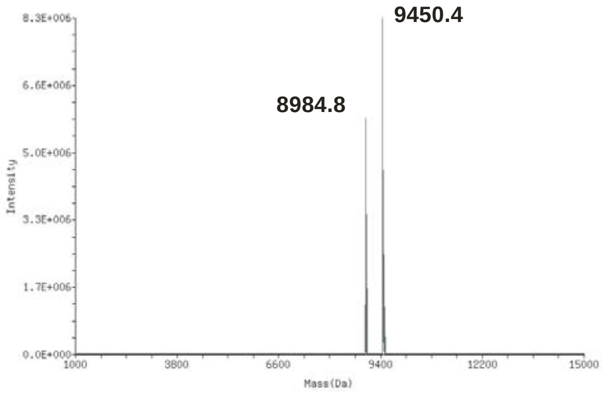


| Sequence | Calc Mol Wt |
|---|---|
| 5′-CTCGTGAGGTGATGcAGGAGATGGGAGGCG | 9449.1 |
| ::::::::::::::::::::::::::::::: | |
| 3′-GAGCACTCCACTACGTCCTCTACCCTCCGC | 8983.8 |

**Duplex S1-rC + *P.a.* RNase H2**

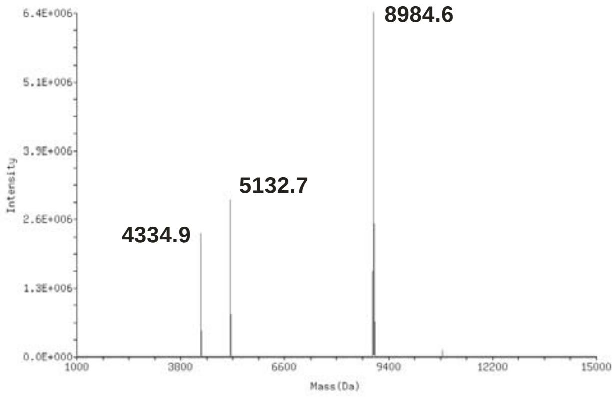


| Sequence | Calc Mol Wt |
|---|---|
| 5′-CTCGTGAGGTGATG-OH | 4334.9 |
| 5′ phos-cAGGAGATGGGAGGCG | 5132.3 |
| 3′-GAGCACTCCACTACGTCCTCTACCCTCCGC | 8983.8 |

**Figure S1. Identification of RNase H2 cleavage products by mass spectrometry.** The synthetic oligonucleotide substrates shown were examined before and after cleavage by recombinant *Pyrococcus abyssi* RNase H2 using electrospray ionization mass spectrometry (ESI-MS). Mass spectra and measured masses are shown to the left. Substrates and reaction products with calculated molecular weights are shown to the right. DNA bases are indicated in black upper case and RNA bases are indicated in red lower case letters.
