# Supplementary material for: RNase H-dependent PCR (rhPCR): improved specificity and single nucleotide polymorphism detection using blocked cleavable primers
Source: BMC Biotechnol. 2011 Aug 10;11:80. doi: 10.1186/1472-6750-11-80 (PMC3224242; doi:10.1186/1472-6750-11-80)
Supplement: Additional File 4 — Figure S2. Mg2+ dependence of P.a. RNase H2 activity. 32P-labeled substrate S-rC 14-1-15 was incubated in the absence or presence of 0.25 mU of recombinant P.a. RNase H2 for 20 minutes at 70°C in Mg Cleavage Buffer (10 mM Tris-HCl pH 8.0, 50 mM NaCl, 10 μg/mL BSA, 0.01% Triton X-100) with varying concentrations of MgCl2 as indicated. Reactions were stopped with the addition of EDTA and cleavage products were separated by denaturing PAGE and visualized by phosphorimaging. The phosphor gel image was quantified and the percent cleavage of substrate (Y-axis) is shown plotted against Mg2+ concentration (X-axis). [file 1472-6750-11-80-S4.PDF]

Additional File 4:

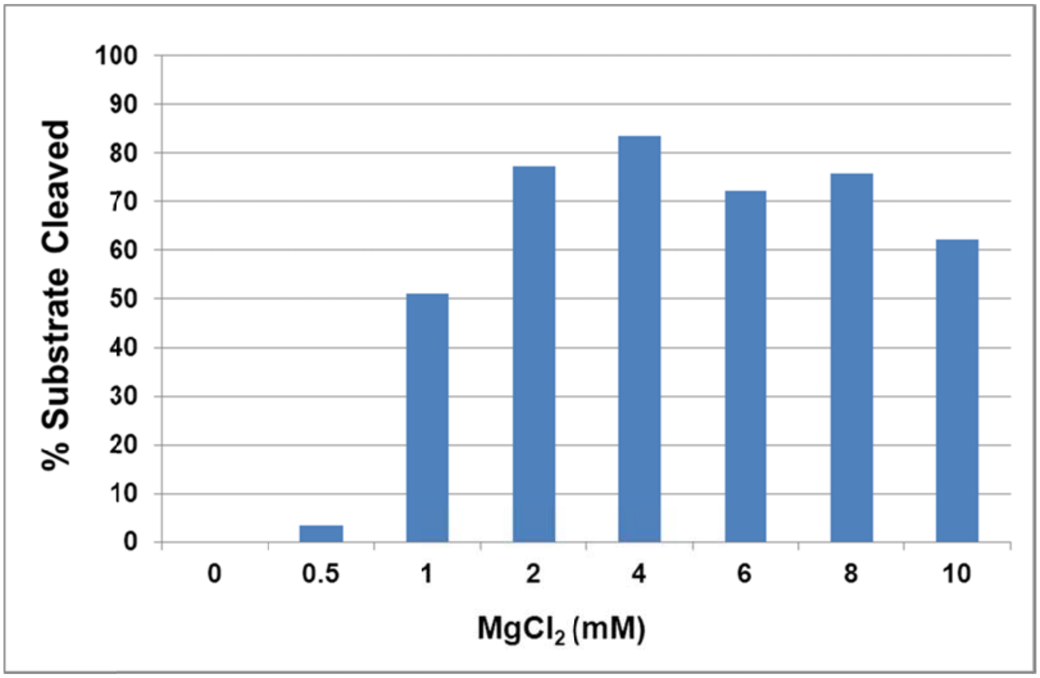

Figure S2. Mg^{2+} dependence of *P.a.* RNase H2 activity.
^{32}P-labeled substrate S-rC 14-1-15 was incubated in the absence or presence of 0.25 mU of recombinant *P.a*. RNase H2 for 20 minutes at 70°C in Mg Cleavage Buffer (10 mM Tris-HCl pH 8.0, 50 mM NaCl, 10 µg/mL BSA, 0.01% Triton X-100) with varying concentrations of $MgCl_2$ as indicated. Reactions were stopped with the addition of EDTA and cleavage products were separated by denaturing PAGE and visualized by phosphorimaging. The phosphor gel image was quantified and the percent cleavage of substrate (Y-axis) is shown plotted against Mg^{2+} concentration (X-axis).
