# Supplementary material for: RNase H-dependent PCR (rhPCR): improved specificity and single nucleotide polymorphism detection using blocked cleavable primers
Source: BMC Biotechnol. 2011 Aug 10;11:80. doi: 10.1186/1472-6750-11-80 (PMC3224242; doi:10.1186/1472-6750-11-80)
Supplement: Additional File 5 — Figure S3. Optimization of primer design for rhPCR. Design of blocked-cleavable primers for use in rhPCR was optimized using a 103 base synthetic oligonucleotide target. A single unmodified Forward (For) primer was used with different blocked-cleavable Reverse (Rev) primers and compared for their relative ability to prime a PCR assay. Blocked-cleavable Rev primers used the same sequence as the unmodified control Rev primer, with the addition of a rU base, and were serially extended by adding 2, 3, 4, 5, or 6 DNA bases 3'-to the ribonucleotide. All blocked primers ended in a ddC residue. Following 45 cycles of PCR, products were separated by denaturing PAGE, fluorescently stained and visualized by UV excitation. M = oligo size markers (bases). [file 1472-6750-11-80-S5.PDF]

Additional File 5:

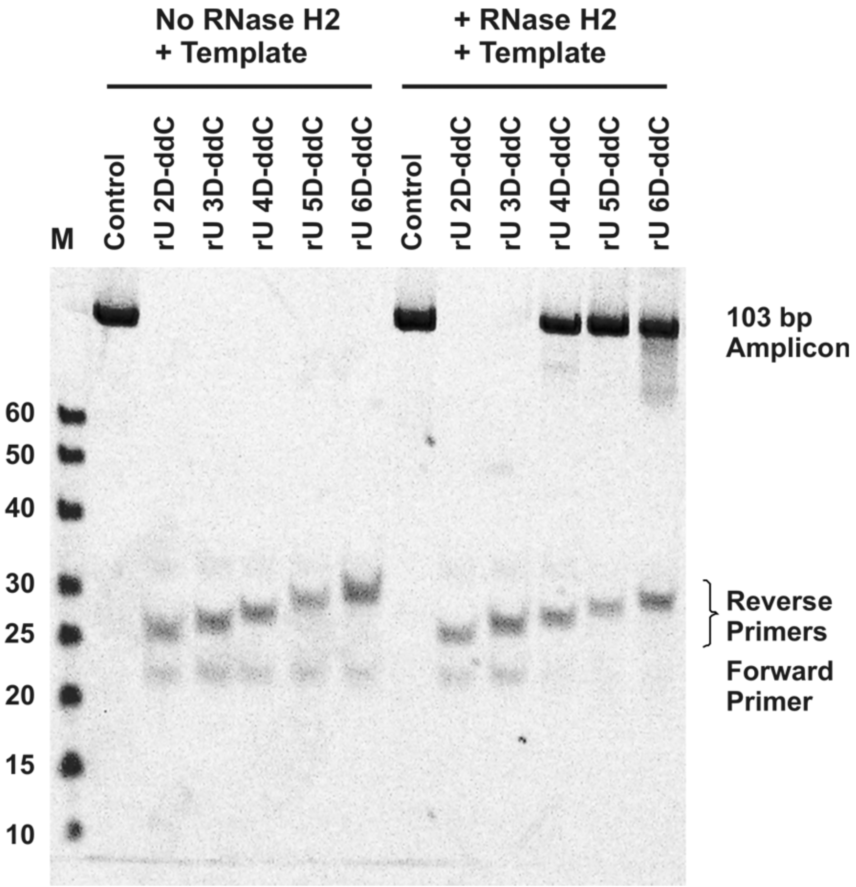

Figure S3. Optimization of primer design for rhPCR.

Design of blocked-cleavable primers for use in rhPCR was optimized using a 103 base synthetic oligonucleotide target. A single unmodified Forward (For) primer was used with different blocked-cleavable Reverse (Rev) primers and compared for their relative ability to prime a PCR assay. Blocked-cleavable Rev primers used the same sequence as the unmodified control Rev primer, with the addition of a rU base, and were serially extended by adding 2, 3, 4, 5, or 6 DNA bases 3′-to the ribonucleotide. All blocked primers ended in a ddC residue. Following 45 cycles of PCR, products were separated by denaturing PAGE, fluorescently stained and visualized by UV excitation. M = oligo size markers (bases).
