# Supplementary material for: RNase H-dependent PCR (rhPCR): improved specificity and single nucleotide polymorphism detection using blocked cleavable primers
Source: BMC Biotechnol. 2011 Aug 10;11:80. doi: 10.1186/1472-6750-11-80 (PMC3224242; doi:10.1186/1472-6750-11-80)
Supplement: Additional File 6 — Figure S4. Mismatch discrimination using rhPCR with the mismatch positioned at the "-1" position relative to the RNA base. Sixteen synthetic oligonucleotide targets were employed where the base complementary to the single RNA residue in the blocked-cleavable primers was fixed (A, C, G, or T) and the base paired opposite position "-1" immediately 5'-to the RNA base in the primer was varied (A, C, G, or T). Likewise a set of 16 "rDDDDx" blocked-cleavable primers was employed where the RNA base was fixed (rA, rC, rG, or rU) and the base at the "-1" position was varied (A, C, G, or T). The target sequence and primers were otherwise the same as in Figure S3, except that the control non-discriminatory primer was one base shorter on the 3'-end. Assay conditions and calculations of ΔCq values were the same as in Figure 7 in the manuscript. All reactions were run in triplicate. [file 1472-6750-11-80-S6.PDF]

**Additional File 6:**

**NrU Blocked Primer** — Template Sequence

| | A | C | G | T |
|---|---|---|---|---|
| A | 16.1 | 8.7 | 12.6 | 0 |
| C | 7.6 | 3.9 | 0 | 12.0 |
| G | 13.8 | 0 | 12.4 | 5.9 |
| T | 0 | 5.2 | 2.4 | 6.2 |

**NrC Blocked Primer** — Template Sequence

| | A | C | G | T |
|---|---|---|---|---|
| A | 13.0 | 8.2 | 10.5 | 0 |
| C | 5.0 | 3.3 | 0 | 3.5 |
| G | 8.3 | 0 | 7.0 | 0.8 |
| T | 0 | 5.4 | 2.1 | 4.6 |

**NrA Blocked Primer** — Template Sequence

| | A | C | G | T |
|---|---|---|---|---|
| A | 14.2 | 8.6 | 11.8 | 0 |
| C | 6.9 | 12.6 | 0 | 6.8 |
| G | 12.8 | 0 | 12.6 | 8.9 |
| T | 0 | 5.1 | 1.4 | 8.6 |

**NrG Blocked Primer** — Template Sequence

| | A | C | G | T |
|---|---|---|---|---|
| A | 12.4 | 4.8 | 10.4 | 0 |
| C | 4.5 | 11.1 | 0 | 2.5 |
| G | 10.3 | 0 | 10.1 | 3.8 |
| T | 0 | 3.5 | 2.2 | 5.3 |

**Figure S4. Mismatch discrimination using rhPCR with the mismatch positioned at the "-1" position relative to the RNA base.**

Sixteen synthetic oligonucleotide targets were employed where the base complementary to the single RNA residue in the blocked-cleavable primers was fixed (A, C, G, or T) and the base paired opposite position "-1" immediately 5′-to the RNA base in the primer was varied (A, C, G, or T). Likewise a set of 16 "rDDDDx" blocked-cleavable primers was employed where the RNA base was fixed (rA, rC, rG, or rU) and the base at the "-1" position was varied (A, C, G, or T). The target sequence and primers were otherwise the same as in Figure 2, except that the control non-discriminatory primer was one base shorter on the 3′-end. Assay conditions and calculations of ΔCq values were the same as in Figure 7 in the manuscript. All reactions were run in triplicate.
