# Supplementary material for: RNase H-dependent PCR (rhPCR): improved specificity and single nucleotide polymorphism detection using blocked cleavable primers
Source: BMC Biotechnol. 2011 Aug 10;11:80. doi: 10.1186/1472-6750-11-80 (PMC3224242; doi:10.1186/1472-6750-11-80)
Supplement: Additional file 7 — Figure S5. Mismatch discrimination using rhPCR with the mismatch positioned at the "+1" position relative to the RNA base. Sixteen synthetic oligonucleotide targets were employed where the base complementary to the single RNA residue in the blocked-cleavable primers was fixed (A, C, G, or T) and the base paired opposite position "+1" immediately 5'-to the RNA base in the primer was varied (A, C, G, or T). Likewise a set of 16 "rDDDDx" blocked-cleavable primers was employed where the RNA base was fixed (rA, rC, rG, or rU) and the base at the "+1" position was varied (A, C, G, or T). The target sequence and primers were otherwise the same as in Figure S3. Assay conditions and calculations of ΔCq values were the same as in Figure 7 in the manuscript. All reactions were run in triplicate. [file 1472-6750-11-80-S7.PDF]

Additional File 7:

rUN Blocked Primer / Template Sequence

	A	C	G	T
A	11.4	2.5	12.2	0
C	6.4	10.4	0	9.0
G	13.8	0	4.6	3.0
T	0	11.1	11.9	2.9

rCN Blocked Primer / Template Sequence

	A	C	G	T
A	5.6	1.8	10.2	0
C	8.8	9.6	0	8.6
G	9.8	0	3.2	0.3
T	0	2.1	0.2	0

rAN Blocked Primer / Template Sequence

	A	C	G	T
A	3.1	1.0	6.1	0
C	9.3	10.2	0	8.3
G	13.2	0	2.5	5.9
T	0	5.0	7.1	4.0

rGN Blocked Primer / Template Sequence

	A	C	G	T
A	6.2	3.0	11.4	0
C	9.5	7.3	0	4.7
G	13.1	0	6.0	3.2
T	0	4.5	11.5	0.3

Figure S5. Mismatch discrimination using rhPCR with the mismatch positioned at the "+1" position relative to the RNA base.
Sixteen synthetic oligonucleotide targets were employed where the base complementary to the single RNA residue in the blocked-cleavable primers was fixed (A, C, G, or T) and the base paired opposite position "+1" immediately 5′-to the RNA base in the primer was varied (A, C, G, or T). Likewise a set of 16 "rDDDDx" blocked-cleavable primers was employed where the RNA base was fixed (rA, rC, rG, or rU) and the base at the "+1" position was varied (A, C, G, or T). The target sequence and primers were otherwise the same as in Figure 2. Assay conditions and calculations of ΔCq values were the same as in Figure 7 in the manuscript. All reactions were run in triplicate.
