# Supplementary material for: RNase H-dependent PCR (rhPCR): improved specificity and single nucleotide polymorphism detection using blocked cleavable primers
Source: BMC Biotechnol. 2011 Aug 10;11:80. doi: 10.1186/1472-6750-11-80 (PMC3224242; doi:10.1186/1472-6750-11-80)
Supplement: Additional File 8 — Table S2. Efficiency of rhPCR at different anneal/extend temperatures. Amplification reactions were run in standard format (10 L reactions with 2.6 mU P.a. RNase H2) using 2-step PCR with anneal/extend temperatures of 50oC, 55oC, and 60oC. The SMAD7 SNP assay and “rDDDDx” blocked-cleavable primers were employed, as in Table 2. [file 1472-6750-11-80-S8.PDF]

Additional File 8:

Primer Sequences	Cq values								
	60°C			55°C			50°C		
	(T/T)	(C/C)	ΔCq	(T/T)	(C/C)	ΔCq	(T/T)	(C/C)	ΔCq
…AA	26.0	26.0	-	26.0	25.7		26.6	26.0	-
…AAcAGGA-x	38.7	26.6	12.1	40.5	26.7	13.8	41.6	27.6	14.0
…AAuAGGA-x	27.9	40.5	12.6	27.6	37.7	10.1	29.3	41.1	11.8

Table S2. Efficiency of rhPCR at different anneal/extend temperatures.
Amplification reactions were run in standard format (10 µL reactions with 2.6 mU *P.a.* RNase H2) using 2-step PCR with anneal/extend temperatures of 50°C, 55°C, and 60°C. The SMAD7 SNP assay and "rDDDDx" blocked-cleavable primers were employed, as in Table 2.
