# Supplementary material for: RNase H-dependent PCR (rhPCR): improved specificity and single nucleotide polymorphism detection using blocked cleavable primers
Source: BMC Biotechnol. 2011 Aug 10;11:80. doi: 10.1186/1472-6750-11-80 (PMC3224242; doi:10.1186/1472-6750-11-80)
Supplement: Additional File 9 — Unified additional materials. All additional files are merged to improve convenience when saving or printing these data. [file 1472-6750-11-80-S9.PDF]

Additional File 9.
Unified set of all Additional Files 1-8.

Additional File 1.
Table S1. Synthetic oligonucleotide sequences

Figure #	Name	Sequence
**Figure S1**	S-rC 14-1-15	**CTCGTGAGGTGATGcAGGAGATGGGAGGCG**
	AS-dC	**CGCCTCCCATCTCCTGCATCACCTCACGAG**
**Figure 2**	S-rC 14-1-15	**CTCGTGAGGTGATGcAGGAGATGGGAGGCG**
	AS-dC	**CGCCTCCCATCTCCTGCATCACCTCACGAG**
**Figure S2**	S-rC 14-1-15	**CTCGTGAGGTGATGcAGGAGATGGGAGGCG**
	AS-dC	**CGCCTCCCATCTCCTGCATCACCTCACGAG**
**Figure 3**	S1RC Sense	**CTCGTGAGGTGATGcAGGAGATGGGAGGCG**
	S-rC 14-1-15 antisense	**CGCCTCCCATCTCCTGCATCACCTCACGAG**
	S-rG 14-1-15 antisense	**CGCCTCCCATCTCCTCCATCACCTCACGAG**
	S-rA 14-1-15 antisense	**CGCCTCCCATCTCCTTCATCACCTCACGAG**
	S-rU 14-1-15 antisense	**CGCCTCCCATCTCCTACATCACCTCACGAG**
	rC AS mismatch -1 T	**CGCCTCCCATCTCCTGTATCACCTCACGAG**
	rC AS mismatch -1 A	**CGCCTCCCATCTCCTGAATCACCTCACGAG**
	rC AS mismatch -1 G	**CGCCTCCCATCTCCTGGATCACCTCACGAG**
	rC AS mismatch -2 G	**CGCCTCCCATCTCCTGCGTCACCTCACGAG**
	rC AS mismatch -2 C	**CGCCTCCCATCTCCTGCCTCACCTCACGAG**
	rC AS mismatch -2 T	**CGCCTCCCATCTCCTGCTTCACCTCACGAG**
	rC AS mismatch -3 G	**CGCCTCCCATCTCCTGCAGCACCTCACGAG**
	rC AS mismatch -3 A	**CGCCTCCCATCTCCTGCAACACCTCACGAG**
	rC AS mismatch -3 C	**CGCCTCCCATCTCCTGCACCACCTCACGAG**
	rC AS mismatch -4 T	**CGCCTCCCATCTCCTGCATTACCTCACGAG**
	rC AS mismatch -4 A	**CGCCTCCCATCTCCTGCATAACCTCACGAG**
	rC AS mismatch -4 G	**CGCCTCCCATCTCCTGCATGACCTCACGAG**
	rC AS mismatch -5 T	**CGCCTCCCATCTCCTGCATCTCCTCACGAG**
	rC AS mismatch -5 G	**CGCCTCCCATCTCCTGCATCGCCTCACGAG**
	rC AS mismatch -5 C	**CGCCTCCCATCTCCTGCATCCCCTCACGAG**
	rC AS mismatch +1 C	**CGCCTCCCATCTCCCGCATCACCTCACGAG**
	rC AS mismatch +1 A	**CGCCTCCCATCTCCAGCATCACCTCACGAG**
	rC AS mismatch +1 G	**CGCCTCCCATCTCCGGCATCACCTCACGAG**

	rC AS mismatch +2 A	CGCCTCCCATCTCATGCATCACCTCACGAG
	rC AS mismatch +2 T	CGCCTCCCATCTCTTGCATCACCTCACGAG
	rC AS mismatch +2 G	CGCCTCCCATCTCGTGCATCACCTCACGAG
	rC AS mismatch +3 G	CGCCTCCCATCTGCTGCATCACCTCACGAG
	rC AS mismatch +3 T	CGCCTCCCATCTTCTGCATCACCTCACGAG
	rC AS mismatch +3 A	CGCCTCCCATCTACTGCATCACCTCACGAG
	rC AS mismatch +4 C	CGCCTCCCATCCCCTGCATCACCTCACGAG
	rC AS mismatch +4 A	CGCCTCCCATCACCTGCATCACCTCACGAG
	rC AS mismatch +4 G	CGCCTCCCATCGCCTGCATCACCTCACGAG
	rC AS mismatch +5 A	CGCCTCCCATATCCTGCATCACCTCACGAG
	rC AS mismatch +5 T	CGCCTCCCATTTCCTGCATCACCTCACGAG
	rC AS mismatch +5 G	CGCCTCCCATGTCCTGCATCACCTCACGAG
**Figure S3**	SynRev 6DrU ddC	CTGAGCTTCATGCCTTTACTGTuCCCCGA/ddC/
	SynRev 5DrU ddC	CTGAGCTTCATGCCTTTACTGTuCCCCG/ddC/
	SynRev 4DrU ddC	CTGAGCTTCATGCCTTTACTGTuCCCC/ddC/
	SynRev 3DrU ddC	CTGAGCTTCATGCCTTTACTGTuCCC/ddC/
	SynRev 3DrU ddC	CTGAGCTTCATGCCTTTACTGTuCC/ddC/
	SynTemp AAG	AGCTCTGCCCAAAGATTACCCTGACAGCTAAGTGGCAGTGGAAGTTGGCCTC AGAAGTAGTGGCCAGCTGTGTGTCGGGGAACAGTAAAGGCATGAAGCTCAG
	SynFor unblocked	AGCTCTGCCCAAAGATTACCCTG
	SynRev non-discrimin	CTGAGCTTCATGCCTTTACTGT
	SynAmp Probe	FAM-TTCTGAGGCCAACTTCCACTGCCACTTA-IBFQ
**Figure 4**	SynFor rA C3 blocked	AGCTCTGCCCAAAGATTACCCTGaCAGC-x
	SynRev 4DrU C3	CTGAGCTTCATGCCTTTACTGTuCCCC-x
	SynTemp AAG	AGCTCTGCCCAAAGATTACCCTGACAGCTAAGTGGCAGTGGAAGTTGGCCTC AGAAGTAGTGGCCAGCTGTGTGTCGGGGAACAGTAAAGGCATGAAGCTCAG
	SynFor unblocked	AGCTCTGCCCAAAGATTACCCTG
	SynRev non-discrimin	CTGAGCTTCATGCCTTTACTGT
	SynAmp Probe	FAM-TTCTGAGGCCAACTTCCACTGCCACTTA-IBFQ
**Figure 5**	Control HCV For	GCAGAAAGCGTCTAGCCATGGCGTTA
	Control HCV Rev	GCAAGCACCCTATCAGGCAGTACCACAA
	HCV 4DrG For	GCAGAAAGCGTCTAGCCATGGCGTTAgTATG-x
	HCV 4DrG Rev	GCAAGCACCCTATCAGGCAGTACCACAAgGCCT-x

	HCV Amplicon	GCAGAAAGCGTCTAGCCATGGCGTTAGTATGAGTGTCGTGCAGCCTCCAGGA<br>CCCCCCCTCCCGGGAGAGCCATAGTGGTCTGCGGAACCGGTGAGTACACCGG<br>AATTGCCAGGACGACCGGGTCCTTTCTTGGACTAAACCCGCTCAATGCCTGG<br>AGATTTGGGCGTGCCCCCGCGAGACTGCTAGCCGAGTAGTGTTGGGTCGCGA<br>AAGGCCTTGTGGTACTGCCTGATAGGGTGCTTGC
**Figure 6**	Control HRAS For	ACCTCGGCCAAGACCC
	Control HRAS Rev	CCTTCCTTCCTTCCTTGCTTCC
	HRAS 4D rG For	ACCTCGGCCAAGACCCgGCAG-x
	HRAS 4D rG Rev	CCTTCCTTCCTTCCTTGCTTCCgTCCT-x
**Figure 7**	SynRev 4DrU C3	CTGAGCTTCATGCCTTTACTGTuCCCC/3SpC3/
	SynRev 4DrA C3	CTGAGCTTCATGCCTTTACTGTaCCCC/3SpC3/
	SynRev 4DrC C3	CTGAGCTTCATGCCTTTACTGTcCCCC/3SpC3/
	SynRev 4DrG C3	CTGAGCTTCATGCCTTTACTGTgCCCC/3SpC3/
	SynRev -T unblocked	CTGAGCTTCATGCCTTTACTGTT
	SynRev -A unblocked	CTGAGCTTCATGCCTTTACTGTA
	SynRev -C unblocked	CTGAGCTTCATGCCTTTACTGTC
	SynRev -G unblocked	CTGAGCTTCATGCCTTTACTGTG
	SynFor unblocked	AGCTCTGCCCAAAGATTACCCTG
	SynRev non-discrimin	CTGAGCTTCATGCCTTTACTGT
	SynTemp AAG	AGCTCTGCCCAAAGATTACCCTGACAGCTAAGTGGCAGTGGAAGTTGGCCTC<br>AGAAGTAGTGGCCAGCTGTGTGTCGGGGAACAGTAAAGGCATGAAGCTCAG
	SynTemp ATG	AGCTCTGCCCAAAGATTACCCTGACAGCTAAGTGGCAGTGGAAGTTGGCCTC<br>AGAAGTAGTGGCCAGCTGTGTGTCGGGGTACAGTAAAGGCATGAAGCTCAG
	SynTemp AGG	AGCTCTGCCCAAAGATTACCCTGACAGCTAAGTGGCAGTGGAAGTTGGCCTC<br>AGAAGTAGTGGCCAGCTGTGTGTCGGGGGACAGTAAAGGCATGAAGCTCAG
	SynTemp ACG	AGCTCTGCCCAAAGATTACCCTGACAGCTAAGTGGCAGTGGAAGTTGGCCTC<br>AGAAGTAGTGGCCAGCTGTGTGTCGGGGCACAGTAAAGGCATGAAGCTCAG
**Figure S4**	SynRev 4DrU C3	CTGAGCTTCATGCCTTTACTGTuCCCC/3SpC3/
	SynRev 4D CrU C3	CTGAGCTTCATGCCTTTACTGCuCCCC/3SpC3/
	SynRev 4D GrU C3	CTGAGCTTCATGCCTTTACTGGuCCCC/3SpC3/
	SynRev 4D ArU C3	CTGAGCTTCATGCCTTTACTGAuCCCC/3SpC3/
	SynRev 4DrA C3	CTGAGCTTCATGCCTTTACTGTaCCCC/3SpC3/
	SynRev 4D CrA C3	CTGAGCTTCATGCCTTTACTGCaCCCC/3SpC3/
	SynRev 4D GrA C3	CTGAGCTTCATGCCTTTACTGGaCCCC/3SpC3/
	SynRev 4D ArA C3	CTGAGCTTCATGCCTTTACTGAaCCCC/3SpC3/
	SynRev 4DrC C3	CTGAGCTTCATGCCTTTACTGTcCCCC/3SpC3/
	SynRev 4D CrC C3	CTGAGCTTCATGCCTTTACTGCcCCCC/3SpC3/
	SynRev 4D GrC C3	CTGAGCTTCATGCCTTTACTGGcCCCC/3SpC3/

	SynRev 4D ArC C3	CTGAGCTTCATGCCTTTACTGAcCCCC/3SpC3/
	SynRev 4DrG C3	CTGAGCTTCATGCCTTTACTGTgCCCC/3SpC3/
	SynRev 4D CrG C3	CTGAGCTTCATGCCTTTACTGCgCCCC/3SpC3/
	SynRev 4D GrG C3	CTGAGCTTCATGCCTTTACTGGgCCCC/3SpC3/
	SynRev 4D ArG C3	CTGAGCTTCATGCCTTTACTGAgCCCC/3SpC3/
	SynRev Short	CTGAGCTTCATGCCTTTACTG
	SynFor unblocked	AGCTCTGCCCAAAGATTACCCTG
	SynTemp AAG	AGCTCTGCCCAAAGATTACCCTGACAGCTAAGTGGCAGTGGAAGTTGGCCTC<br>AGAAGTAGTGGCCAGCTGTGTGTCGGGGAACAGTAAAGGCATGAAGCTCAG
	SynTemp TAG	AGCTCTGCCCAAAGATTACCCTGACAGCTAAGTGGCAGTGGAAGTTGGCCTC<br>AGAAGTAGTGGCCAGCTGTGTGTCGGGGATCAGTAAAGGCATGAAGCTCAG
	SynTemp CAG	AGCTCTGCCCAAAGATTACCCTGACAGCTAAGTGGCAGTGGAAGTTGGCCTC<br>AGAAGTAGTGGCCAGCTGTGTGTCGGGGACCAGTAAAGGCATGAAGCTCAG
	SynTemp GAG	AGCTCTGCCCAAAGATTACCCTGACAGCTAAGTGGCAGTGGAAGTTGGCCTC<br>AGAAGTAGTGGCCAGCTGTGTGTCGGGGAGCAGTAAAGGCATGAAGCTCAG
	SynTemp ATG	AGCTCTGCCCAAAGATTACCCTGACAGCTAAGTGGCAGTGGAAGTTGGCCTC<br>AGAAGTAGTGGCCAGCTGTGTGTCGGGGTACAGTAAAGGCATGAAGCTCAG
	SynTemp TTG	AGCTCTGCCCAAAGATTACCCTGACAGCTAAGTGGCAGTGGAAGTTGGCCTC<br>AGAAGTAGTGGCCAGCTGTGTGTCGGGGTTCAGTAAAGGCATGAAGCTCAG
	SynTemp CTG	AGCTCTGCCCAAAGATTACCCTGACAGCTAAGTGGCAGTGGAAGTTGGCCTC<br>AGAAGTAGTGGCCAGCTGTGTGTCGGGGTCCAGTAAAGGCATGAAGCTCAG
	SynTemp GTG	AGCTCTGCCCAAAGATTACCCTGACAGCTAAGTGGCAGTGGAAGTTGGCCTC<br>AGAAGTAGTGGCCAGCTGTGTGTCGGGGTGCAGTAAAGGCATGAAGCTCAG
	SynTemp AGG	AGCTCTGCCCAAAGATTACCCTGACAGCTAAGTGGCAGTGGAAGTTGGCCTC<br>AGAAGTAGTGGCCAGCTGTGTGTCGGGGGACAGTAAAGGCATGAAGCTCAG
	SynTemp TGG	AGCTCTGCCCAAAGATTACCCTGACAGCTAAGTGGCAGTGGAAGTTGGCCTC<br>AGAAGTAGTGGCCAGCTGTGTGTCGGGGGTCAGTAAAGGCATGAAGCTCAG
	SynTemp CGG	AGCTCTGCCCAAAGATTACCCTGACAGCTAAGTGGCAGTGGAAGTTGGCCTC<br>AGAAGTAGTGGCCAGCTGTGTGTCGGGGGCCAGTAAAGGCATGAAGCTCAG
	SynTemp GGG	AGCTCTGCCCAAAGATTACCCTGACAGCTAAGTGGCAGTGGAAGTTGGCCTC<br>AGAAGTAGTGGCCAGCTGTGTGTCGGGGGGCAGTAAAGGCATGAAGCTCAG
	SynTemp ACG	AGCTCTGCCCAAAGATTACCCTGACAGCTAAGTGGCAGTGGAAGTTGGCCTC<br>AGAAGTAGTGGCCAGCTGTGTGTCGGGGCACAGTAAAGGCATGAAGCTCAG
	SynTemp TCG	AGCTCTGCCCAAAGATTACCCTGACAGCTAAGTGGCAGTGGAAGTTGGCCTC<br>AGAAGTAGTGGCCAGCTGTGTGTCGGGGCTCAGTAAAGGCATGAAGCTCAG
	SynTemp CCG	AGCTCTGCCCAAAGATTACCCTGACAGCTAAGTGGCAGTGGAAGTTGGCCTC<br>AGAAGTAGTGGCCAGCTGTGTGTCGGGGCCCAGTAAAGGCATGAAGCTCAG
	SynTemp GCG	AGCTCTGCCCAAAGATTACCCTGACAGCTAAGTGGCAGTGGAAGTTGGCCTC<br>AGAAGTAGTGGCCAGCTGTGTGTCGGGGCGCAGTAAAGGCATGAAGCTCAG
**Figure S5**	SynRev 4DrU C3	CTGAGCTTCATGCCTTTACTGTuCCCC/3SpC3/
	SynRev 4D rUA C3	CTGAGCTTCATGCCTTTACTGTuACCC/3SpC3/

	SynRev 4D rUT C3	CTGAGCTTCATGCCTTTACTGTuTCCC/3SpC3/
	SynRev 4D rUG C3	CTGAGCTTCATGCCTTTACTGTuGCCC/3SpC3/
	SynRev 4DrA C3	CTGAGCTTCATGCCTTTACTGTaCCCC/3SpC3/
	SynRev 4D rAA C3	CTGAGCTTCATGCCTTTACTGTaACCC/3SpC3/
	SynRev 4D rAT C3	CTGAGCTTCATGCCTTTACTGTaTCCC/3SpC3/
	SynRev 4D rAG C3	CTGAGCTTCATGCCTTTACTGTaGCCC/3SpC3/
	SynRev 4DrC C3	CTGAGCTTCATGCCTTTACTGTcCCCC/3SpC3/
	SynRev 4D rCA C3	CTGAGCTTCATGCCTTTACTGTcACCC/3SpC3/
	SynRev 4D rCT C3	CTGAGCTTCATGCCTTTACTGTcTCCC/3SpC3/
	SynRev 4D rCG C3	CTGAGCTTCATGCCTTTACTGTcGCCC/3SpC3/
	SynRev 4DrG C3	CTGAGCTTCATGCCTTTACTGTgCCCC/3SpC3/
	SynRev 4D rGA C3	CTGAGCTTCATGCCTTTACTGTgACCC/3SpC3/
	SynRev 4D rGT C3	CTGAGCTTCATGCCTTTACTGTgTCCC/3SpC3/
	SynRev 4D rGG C3	CTGAGCTTCATGCCTTTACTGTgGCCC/3SpC3/
	SynFor unblocked	AGCTCTGCCCAAAGATTACCCTG
	SynRev non-discrimin	CTGAGCTTCATGCCTTTACTGT
	SynTemp AAG	AGCTCTGCCCAAAGATTACCCTGACAGCTAAGTGGCAGTGGAAGTTGGCCTC AGAAGTAGTGGCCAGCTGTGTGTCGGGGAACAGTAAAGGCATGAAGCTCAG
	SynTemp AAT	AGCTCTGCCCAAAGATTACCCTGACAGCTAAGTGGCAGTGGAAGTTGGCCTC AGAAGTAGTGGCCAGCTGTGTGTCGGGTAACAGTAAAGGCATGAAGCTCAG
	SynTemp AAA	AGCTCTGCCCAAAGATTACCCTGACAGCTAAGTGGCAGTGGAAGTTGGCCTC AGAAGTAGTGGCCAGCTGTGTGTCGGGAAACAGTAAAGGCATGAAGCTCAG
	SynTemp AAC	AGCTCTGCCCAAAGATTACCCTGACAGCTAAGTGGCAGTGGAAGTTGGCCTC AGAAGTAGTGGCCAGCTGTGTGTCGGGCAACAGTAAAGGCATGAAGCTCAG
	SynTemp ATG	AGCTCTGCCCAAAGATTACCCTGACAGCTAAGTGGCAGTGGAAGTTGGCCTC AGAAGTAGTGGCCAGCTGTGTGTCGGGGTACAGTAAAGGCATGAAGCTCAG
	SynTemp ATT	AGCTCTGCCCAAAGATTACCCTGACAGCTAAGTGGCAGTGGAAGTTGGCCTC AGAAGTAGTGGCCAGCTGTGTGTCGGGTTACAGTAAAGGCATGAAGCTCAG
	SynTemp ATA	AGCTCTGCCCAAAGATTACCCTGACAGCTAAGTGGCAGTGGAAGTTGGCCTC AGAAGTAGTGGCCAGCTGTGTGTCGGGATACAGTAAAGGCATGAAGCTCAG
	SynTemp ATC	AGCTCTGCCCAAAGATTACCCTGACAGCTAAGTGGCAGTGGAAGTTGGCCTC AGAAGTAGTGGCCAGCTGTGTGTCGGGCTACAGTAAAGGCATGAAGCTCAG
	SynTemp AGG	AGCTCTGCCCAAAGATTACCCTGACAGCTAAGTGGCAGTGGAAGTTGGCCTC AGAAGTAGTGGCCAGCTGTGTGTCGGGGGACAGTAAAGGCATGAAGCTCAG
	SynTemp AGT	AGCTCTGCCCAAAGATTACCCTGACAGCTAAGTGGCAGTGGAAGTTGGCCTC AGAAGTAGTGGCCAGCTGTGTGTCGGGTGACAGTAAAGGCATGAAGCTCAG
	SynTemp AGA	AGCTCTGCCCAAAGATTACCCTGACAGCTAAGTGGCAGTGGAAGTTGGCCTC AGAAGTAGTGGCCAGCTGTGTGTCGGGAGACAGTAAAGGCATGAAGCTCAG

	SynTemp AGC	AGCTCTGCCCAAAGATTACCCTGACAGCTAAGTGGCAGTGGAAGTTGGCCTC<br>AGAAGTAGTGGCCAGCTGTGTGTCGGGCGACAGTAAAGGCATGAAGCTCAG
	SynTemp ACG	AGCTCTGCCCAAAGATTACCCTGACAGCTAAGTGGCAGTGGAAGTTGGCCTC<br>AGAAGTAGTGGCCAGCTGTGTGTCGGGGCACAGTAAAGGCATGAAGCTCAG
	SynTemp ACT	AGCTCTGCCCAAAGATTACCCTGACAGCTAAGTGGCAGTGGAAGTTGGCCTC<br>AGAAGTAGTGGCCAGCTGTGTGTCGGGTCACAGTAAAGGCATGAAGCTCAG
	SynTemp ACA	AGCTCTGCCCAAAGATTACCCTGACAGCTAAGTGGCAGTGGAAGTTGGCCTC<br>AGAAGTAGTGGCCAGCTGTGTGTCGGGACACAGTAAAGGCATGAAGCTCAG
	SynTemp ACC	AGCTCTGCCCAAAGATTACCCTGACAGCTAAGTGGCAGTGGAAGTTGGCCTC<br>AGAAGTAGTGGCCAGCTGTGTGTCGGGCCACAGTAAAGGCATGAAGCTCAG
**Table 2**	rs4939827 Rev unblocked	CTCACTCTAAACCCCAGCATT
	rs4939827 For non-discr	CAGCCTCATCCAAAAGAGGAAA
	rs4939827 For T	CAGCCTCATCCAAAAGAGGAAAT
	rs4939827 For C	CAGCCTCATCCAAAAGAGGAAAC
	rs4939827 4DrC C3	CAGCCTCATCCAAAAGAGGAAAcAGGA/3SpC3/
	rs4939827 4DrU C3	CAGCCTCATCCAAAAGAGGAAAuAGGA/3SpC3/
	rs4939827 For TA	CAGCCTCATCCAAAAGAGGAAATA
	rs4939827 For CA	CAGCCTCATCCAAAAGAGGAAACA
	rs4939827 4DrAC C3	CAGCCTCATCCAAAAGAGGAAaCAGG/3SpC3/
	rs4939827 4DrAT C3	CAGCCTCATCCAAAAGAGGAAaTAGG/3SpC3/
	rs4939827 4DCrA C3	CAGCCTCATCCAAAAGAGGAAACaGGAC/3SpC3/
	rs4939827 4DTrA C3	CAGCCTCATCCAAAAGAGGAAATaGGAC/3SpC3/
**Table S2**	rs4939827 Rev unblocked	CTCACTCTAAACCCCAGCATT
	rs4939827 For non-discr	CAGCCTCATCCAAAAGAGGAAA
	rs4939827 4DrC C3	CAGCCTCATCCAAAAGAGGAAAcAGGA/3SpC3/
	rs4939827 4DrU C3	CAGCCTCATCCAAAAGAGGAAAuAGGA/3SpC3/
**Figure 8**	rs4939827 Rev unblocked	CTCACTCTAAACCCCAGCATT
	rs4939827 For non-discr	CAGCCTCATCCAAAAGAGGAAA
	rs4939827 4DrC C3	CAGCCTCATCCAAAAGAGGAAAcAGGA/3SpC3/
	rs4939827 4DrU C3	CAGCCTCATCCAAAAGAGGAAAuAGGA/3SpC3/

DNA bases are black uppercase. RNA bases are red lowercase. IBFQ is Iowa Black^{TM}-FQ dark quencher. FAM is 6-carboxyfluorescein. ddC is dideoxycytosine. SpC3 is a C3 propanediol spacer. Locations of mismatched bases relative to the target nucleic acid are underlined. Sequences are shown 5N to 3N.

Additional File 2.
Additional methods: Cloning and characterization of *Pyrococcus abyssi* RNase H2

Sequences of RNase H2 (*rnhb*) genes

Sequence of the codon optimized synthetic gene employed to express recombinant *Pyrococcus abyssi* RNase H2 protein is provided below. Standard codon usage tables for *E. coli* were used. DNA sequence identity was verified on both strands. Lower case letters represent vector sequences, including a Bam HI site on the 5N-end and a Hind III site on the 3N-end used for cloning the gene into the expression plasmid pET-27b(+) (Novagen, Madison, WI). Upper case letters represent coding sequences of the RNase H2 enzyme. The endogenous ATG start codon of the RNase H2 gene is underlined (translation starts upstream of this site in the vector).

Codon optimized *rnhb* gene from *Pyrococcus abyssi*

```
ggatccgATGAAAGTTGCAGGTGCAGATGAAGCTGGTCGTGGTCCAGTTATTGGTCCGCTGGTT
ATTGTTGCTGCTGTTGTGGAGGAAGACAAAATCCGCTCTCTGACTAAGCTGGGTGTTAAAGACT
CCAAACAGCTGACCCCGGCGCAACGTGAAAAACTGTTCGATGAAATCGTAAAAGTACTGGATGA
TTACTCTGTGGTCATTGTGTCCCCGCAGGACATTGACGGTCGTAAGGGCAGCATGAACGAACTG
GAGGTAGAAAACTTCGTTAAAGCCCTGAATAGCCTGAAAGTTAAGCCGGAAGTTATTTACATTG
ATTCCGCTGATGTTAAAGCTGAACGTTTCGCTGAAAACATTCGCAGCCGTCTGGCGTACGAAGC
GAAAGTTGTAGCCGAACATAAAGCGGATGCGAAGTATGAGATCGTATCCGCAGCCTCTATCCTG
GCAAAAGTTATCCGTGACCGCGAGATCGAAAAGCTGAAAGCCGAATACGGTGATTTTGGTTCCG
GTTACCCGTCTGATCCGCGTACTAAGAAATGGCTGGAAGAATGGTATAGCAAACACGGCAATTT
CCCGCCGATCGTGCGTCGTACTTGGGATACTGCAAAGAAAATCGAAGAAAAATTCAAACGTGCG
CAGCTGACCCTGGACAACTTCCTGAAGCGTTTTCGCAACaagctt
```

Production and purification of *P.a.* RNase H2

Several preparations of the enzyme were made. The following protocol is optimized and represents the large scale prep which provided enzyme for the bulk of the studies performed here. BL21(DE3) bacterial cells were transformed with a pET-27b(+) plasmid containing the *E. coli* codon-optimized *Pyrococcus abyssi* RNase H2 gene cloned into the plasmid at the BamHI/HindIII sites. Two x 1 L bacterial cultures were grown to log phase in selective LB media. RNase H2 protein production was induced with 1 mM IPTG at 37°C for 6 hours. Cells were harvested at 5,000 rpm for 10 minutes in a Beckman JLA 10.500 rotor and frozen overnight at -20°C. Cell paste was thawed and 50 mL of Bugbuster® Protein Extraction Reagent, 50 kU rLysozyme™ (Novagen), and 2500 U RNase-free DNase I (Roche, Mannheim, Germany) were added per liter of original culture. Cell lysate was incubated with rotation at 25°C for 30 minutes. The lysate was centrifuged at 16,000 x g for 30 minutes to pellet insoluble materials, and the soluble supernatant was removed and placed in a fresh tube. DNase I was heat inactivated at 75°C for 15 minutes and insoluble materials were removed by centrifugation at 16,000 x g for 10 minutes. A 4% to 20% SDS-polyacrylamide gel was run with increasing amounts of soluble and insoluble material and Coomassie stained to estimate the quantity of RNase H2 present in both fractions. The heat treatment was found to result in a very effective first step of purification; this material was further purified by capture using a His•Bind® column. Elution was performed by 2 x 6 volumes of elution buffer containing 200 mM imidazole. A 70%

ammonium sulfate precipitation was performed to concentrate the purified protein. SDS-PAGE revealed a single band of the expected molecular weight (27.6 kDa) with little contaminating material. The enzyme was dialyzed into Buffer A (10 mM Tris pH 8.0, 1 mM EDTA, 100 mM NaCl, 0.1 % Triton X-100, and 50% glycerol) and stored at -20°C.

Additional File 3.

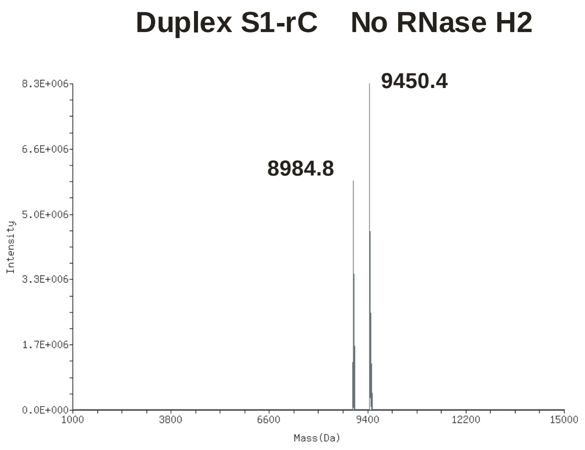

Sequence	Calc Mol Wt
5′-CTCGTGAGGTGATGcAGGAGATGGGAGGCG	9449.1
::::::::::::::::::::::::::::::	
3′-GAGCACTCCACTACGTCCTCTACCCTCCGC	8983.8

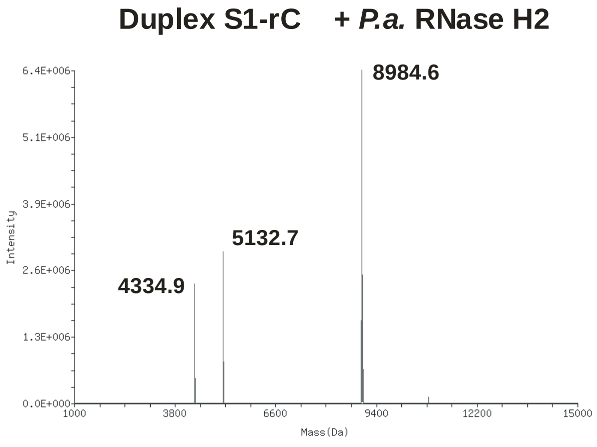

Sequence	Calc Mol Wt
5′-CTCGTGAGGTGATG-OH	4334.9
5′ phos-cAGGAGATGGGAGGCG	5132.3
3′-GAGCACTCCACTACGTCCTCTACCCTCCGC	8983.8

Figure S1. Identification of RNase H2 cleavage products by mass spectrometry. The synthetic oligonucleotide substrates shown were examined before and after cleavage by recombinant *Pyrococcus abyssi* RNase H2 using electrospray ionization mass spectrometry (ESI-MS). Mass spectra and measured masses are shown to the left. Substrates and reaction products with calculated molecular weights are shown to the right. DNA bases are indicated in black upper case and RNA bases are indicated in red lower case letters.

Additional File 4.

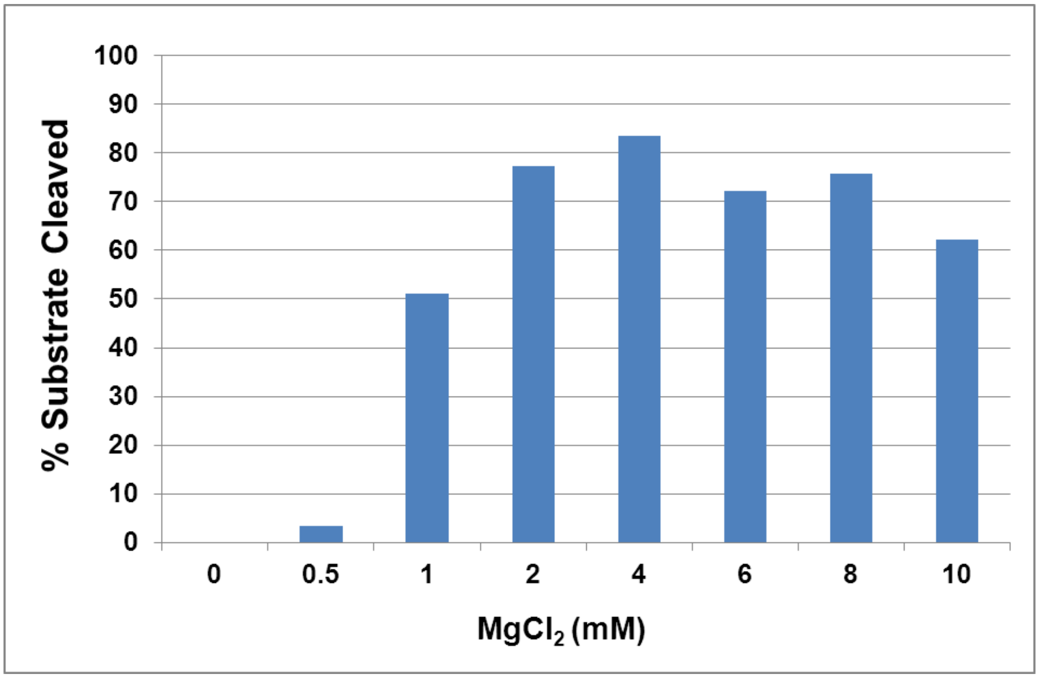

Figure S2. Mg^{2+} dependence of *P.a.* RNase H2 activity.
^{32}P-labeled substrate S-rC 14-1-15 was incubated in the absence or presence of 0.25 mU of recombinant *P.a*. RNase H2 for 20 minutes at 70°C in Mg Cleavage Buffer (10 mM Tris-HCl pH 8.0, 50 mM NaCl, 10 µg/mL BSA, 0.01% Triton X-100) with varying concentrations of $MgCl_2$ as indicated. Reactions were stopped with the addition of EDTA and cleavage products were separated by denaturing PAGE and visualized by phosphorimaging. The phosphor gel image was quantified and the percent cleavage of substrate (Y-axis) is shown plotted against Mg^{2+} concentration (X-axis).

Additional File 5.

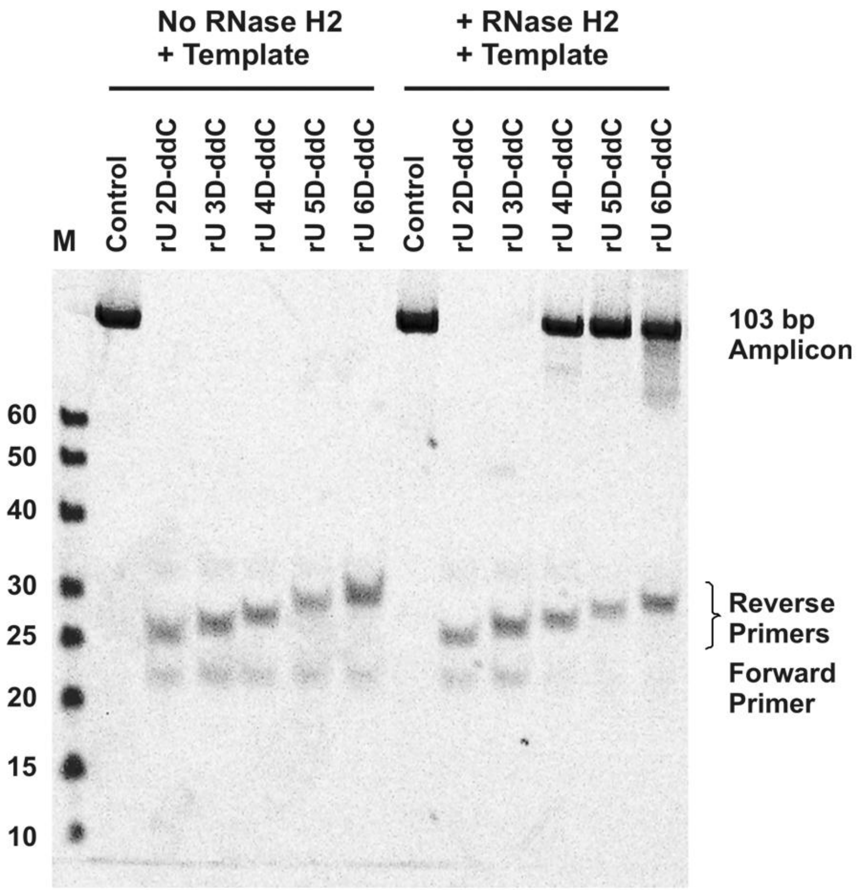

Figure S3. Optimization of primer design for rhPCR.

Design of blocked-cleavable primers for use in rhPCR was optimized using a 103 base synthetic oligonucleotide target. A single unmodified Forward (For) primer was used with different blocked-cleavable Reverse (Rev) primers and compared for their relative ability to prime a PCR assay. Blocked-cleavable Rev primers used the same sequence as the unmodified control Rev primer, with the addition of a rU base, and were serially extended by adding 2, 3, 4, 5, or 6 DNA bases 3N-to the ribonucleotide. All blocked primers ended in a ddC residue. Following 45 cycles of PCR, products were separated by denaturing PAGE, fluorescently stained and visualized by UV excitation. M = oligo size markers (bases).

Additional File 6.

NrU Blocked Primer / Template Sequence

	A	C	G	T
A	16.1	8.7	12.6	0
C	7.6	3.9	0	12.0
G	13.8	0	12.4	5.9
T	0	5.2	2.4	6.2

NrC Blocked Primer / Template Sequence

	A	C	G	T
A	13.0	8.2	10.5	0
C	5.0	3.3	0	3.5
G	8.3	0	7.0	0.8
T	0	5.4	2.1	4.6

NrA Blocked Primer / Template Sequence

	A	C	G	T
A	14.2	8.6	11.8	0
C	6.9	12.6	0	6.8
G	12.8	0	12.6	8.9
T	0	5.1	1.4	8.6

NrG Blocked Primer / Template Sequence

	A	C	G	T
A	12.4	4.8	10.4	0
C	4.5	11.1	0	2.5
G	10.3	0	10.1	3.8
T	0	3.5	2.2	5.3

Figure S4. Mismatch discrimination using rhPCR with the mismatch positioned at the "-1" position relative to the RNA base.

Sixteen synthetic oligonucleotide targets were employed where the base complementary to the single RNA residue in the blocked-cleavable primers was fixed (A, C, G, or T) and the base paired opposite position "-1" immediately 5N-to the RNA base in the primer was varied (A, C, G, or T). Likewise a set of 16 "rDDDDx" blocked-cleavable primers was employed where the RNA base was fixed (rA, rC, rG, or rU) and the base at the "-1" position was varied (A, C, G, or T). The target sequence and primers were otherwise the same as in Figure S3, except that the control non-discriminatory primer was one base shorter on the 3N-end. Assay conditions and calculations of ΔCq values were the same as in Figure 7 in the manuscript. All reactions were run in triplicate.

Additional File 7.

rUN Blocked Primer (rows) / **Template Sequence** (columns)

	A	C	G	T
A	11.4	2.5	12.2	0
C	6.4	10.4	0	9.0
G	13.8	0	4.6	3.0
T	0	11.1	11.9	2.9

rCN Blocked Primer (rows) / **Template Sequence** (columns)

	A	C	G	T
A	5.6	1.8	10.2	0
C	8.8	9.6	0	8.6
G	9.8	0	3.2	0.3
T	0	2.1	0.2	0

rAN Blocked Primer (rows) / **Template Sequence** (columns)

	A	C	G	T
A	3.1	1.0	6.1	0
C	9.3	10.2	0	8.3
G	13.2	0	2.5	5.9
T	0	5.0	7.1	4.0

rGN Blocked Primer (rows) / **Template Sequence** (columns)

	A	C	G	T
A	6.2	3.0	11.4	0
C	9.5	7.3	0	4.7
G	13.1	0	6.0	3.2
T	0	4.5	11.5	0.3

Figure S5. Mismatch discrimination using rhPCR with the mismatch positioned at the "+1" position relative to the RNA base.

Sixteen synthetic oligonucleotide targets were employed where the base complementary to the single RNA residue in the blocked-cleavable primers was fixed (A, C, G, or T) and the base paired opposite position "+1" immediately 5N-to the RNA base in the primer was varied (A, C, G, or T). Likewise a set of 16 "rDDDDx" blocked-cleavable primers was employed where the RNA base was fixed (rA, rC, rG, or rU) and the base at the "+1" position was varied (A, C, G, or T). The target sequence and primers were otherwise the same as in Figure S3. Assay conditions and calculations of ΔCq values were the same as in Figure 7 in the manuscript. All reactions were run in triplicate.

Additional File 8.

Primer Sequences	Cq values								
	60°C			55°C			50°C		
	(T/T)	(C/C)	ΔCq	(T/T)	(C/C)	ΔCq	(T/T)	(C/C)	ΔCq
…AA	26.0	26.0	-	26.0	25.7		26.6	26.0	-
…AAcAGGA-x	38.7	26.6	12.1	40.5	26.7	13.8	41.6	27.6	14.0
…AAuAGGA-x	27.9	40.5	12.6	27.6	37.7	10.1	29.3	41.1	11.8

Table S2. Efficiency of rhPCR at different anneal/extend temperatures.
Amplification reactions were run in standard format (10 μL reactions with 2.6 mU *P.a.* RNase H2) using 2-step PCR with anneal/extend temperatures of 50°C, 55°C, and 60°C. The SMAD7 SNP assay and "rDDDDx" blocked-cleavable primers were employed, as in Table 2.
